# On the Performance Analysis of Switched Diversity Combining Receivers over Fisher–Snedecor ℱ Composite Fading Channels

**DOI:** 10.3390/s21093014

**Published:** 2021-04-25

**Authors:** Weijun Cheng, Xiaoting Wang, Tengfei Ma, Gang Wang

**Affiliations:** 1 School of Information Engineering, Minzu University of China, Beijing 100081, China; 19301542@muc.edu.cn (X.W.); 20301834@muc.edu.cn (T.M.); 2 Key Laboratory of Mining Disaster Prevention and Control, Shandong University of Science and Technology, Qingdao 266590, China; gang.wang@sdust.edu.cn

**Keywords:** switch-and-stay combining, switch-and-examine combining, Fisher–Snedecor ℱ distribution, performance optimization, bivariate statistical characteristics

## Abstract

In some emerging wireless applications, such as wearable communication and low-power sensor network applications, wireless devices or nodes not only require simple physical implementation approaches but also require certain reliable receiver techniques to overcome the effects of multipath or shadowed fading. Switched diversity combining (SDC) systems could be a simple and promising solution to the above requirements. Recently, a Fisher–Snedecor F composited fading model has gained much interest because of its modeling accuracy and calculation tractability. However, the performance of SDC systems over F fading channels has not yet been analyzed in the open literature. To this end, this paper presents a systematic analysis of SDC systems over F fading channels, including dual-branch switch-and-stay combining (SSC), multibranch switch-and examine combining (SEC), and SEC with post-examining selection (SECps) systems. We first investigate the statistical characteristics of univariate and bivariate F distributions. Then, these statistical expressions are introduced into the above SDC systems and the statistical metrics of the output signal-to-noise ratio (SNR) for these systems are deduced in different F fading scenarios. Thirdly, certain exact and novel expressions of performance criteria, such as the outage probability, the average bit error probability and average symbol error probability, as well as the average channel capacity for SSC, SEC, and SECps are derived. To find the optimum performance, optimal analysis is performed for the independent and identically distributed cases. Finally, numerical evaluation and simulations are carried out to demonstrate the validity of the theoretical analysis under various F fading scenarios. According to the obtained results, the multipath fading parameter has more influence on the performance of SDC systems than the shadowing parameter, the correlation coefficient, or the average SNR. Importantly, the SDC systems can provide switched diversity gains only when the switching threshold is not too large or too small compared to the average SNR.

## 1. Introduction

In most traditional wireless communication systems, such as cellular networks and satellite communications, the diversity combining technique is one of the well-known and effective measures to mitigate the adverse effects of fading and to improve system performance and reliability [1]. Among the classical diversity combining techniques, gain diversity combining schemes, namely equal gain combining and maximum ratio combining (MRC), can achieve better system performance compared with selection combining schemes, including pure selection combining (PSC) and switched diversity combining (SDC) [2]. These gain combing schemes and PSC not only need to continually detect all diversity branches but also require some or all knowledge of the channel state information, resulting in a more complex system structure and energy consumption, whereas the advantage of the SDC scheme is that the receiver monitors only one branch at a time and switches to the next branch when the input signal-to-noise ratio (SNR) of the current branch falls below a threshold SNR.

Nowadays, the SDC scheme has three different models, namely dual-branch switch-and-stay combining (SSC) [1], multibranch switch-and examine combining (SEC) [3], and SEC with post-examining selection (SECps) [4]. In an SSC scheme, the receiver selects a particular antenna branch until its instantaneous SNR falls below a predetermined threshold. Whenever this occurs, the receiver switches to the other branch and stays there, regardless of the quality of the other branch. For an SEC scheme, the receiver chooses the first branch and estimates its instantaneous SNR at the beginning, then compares it with a predetermined threshold. If the SNR of the first branch is not less than the predetermined threshold, then the receiver employs the first branch for signal reception. Otherwise, the receiver switches to the second branch and examines it. This process continues until the receiver finds an acceptable branch. If no branch is available, then the receiver usually stays with the last examined branch. The SECps is a modified version of SEC, and the significant difference between SEC and SECps is that the SECps receiver chooses the branch with the maximum SNR instead of the last examined one in SEC, only when all estimated branches are unacceptable. Thus, the SECps can be considered as PSC with an output threshold.

To this end, the performance of SSC, SEC, and SECps for conventional wireless communications applications has been widely investigated over different fading environments in the past decades. However, most studies have focused on multipath fading environments, such as independent Rayleigh [1,3,4,5], Rician [1,3,4,5], Nakagami-m [1,3,4,5,6], generalized gamma [7], κ-μ [8], η-μ [8,9], α-μ [10], correlated Nakagami-m [11], and correlated α-μ [12] environments. Additionally, only a few papers considered shadowing channels and gamma-based composite fading channels for SSC schemes, such as correlated log-normal [13], Rayleigh–gamma [14], generalized K [15], κ-μ–gamma [16], and α-κ-μ–gamma [17] channels. 

Moreover, in some emerging wireless communications applications, such as wireless body area networks and low-cost Internet of Things, SDC schemes have gained more attention again because of their simple structure and ease of implementation. For indoor off-body communication [2] and outdoor wearable communication [18], the authors considered SSC, SEC, and SECps to improve signal reliability over Nakagami-m fading channels. For non-line-of-sight ultra-ultraviolet communication in [19], an SSC diversity reception was presented to overcome the fluctuations caused by strong atmospheric turbulence in a gamma–gamma condition. After this, they further applied the SSC scheme to a blind-spectrum sensing system for multiuser ultraviolet communication in [20]. To enable low-power, ultra-reliable, low-latency downlink communications to low-cost Internet of Things devices in [21], PSC and SSC schemes were involved as simple and promising solutions in Nakagami-m fading channels. In [22], the authors studied the impacts of inter-branch correlation on multichannel spectrum sensing by using PSC and SSC schemes in dual arbitrarily correlated Nakagami-m fading channels. For indoor millimeter-wave communications, the performance of SSC and SEC was analyzed in [23]. In [24], a SECps-based scheduling algorithm was designed for multiuser downlink wiretap networks. The authors in [25] applied PSC and SSC in mobile edge computing networks to choose computational access points over Nakagami-m fading channels. In [26], a distributed SSC receiver was designed for free space optical communications. The authors in [27] studied a SECps scheduling scheme for multirelay networks in shadowed Rician channels. However, in the above new wireless applications, only the impact of multipath fading on SSC, SEC, and SECps models is considered and the influence of the shadowing fading is still ignored, except in the method proposed in [27]. As a matter of fact, the composite fading effects combing multipath and shadowing effects are frequently encountered in emerging wireless communications scenarios, such as body area networks [2] and vehicle-to-vehicle applications [28]. In Table 1, we summarize the applications of the SDC schemes for new wireless communications systems.

More recently, to achieve an appropriate balance between modeling accuracy and calculation complexity, a Fisher–Snedecor F composite fading channel model was proposed in [29]. It accurately characterizes the composite effects of both small- and large-scale fading, in which the small-scale multipath fading follows the Nakagami-m distribution, whereas the large-scale shadowing fading follows an inverse Nakagami-m distribution. Compared to the well-known generalized K composite fading model, this model not only provides more tractable and simpler mathematical formulations of the statistical characteristics but also shows a better fit to experimental channel measurements in some emerging wireless communications applications, for example device-to-device communications and wireless body area networks. As a result, a good deal of research work has been done based on the Fisher–Snedecor F composite fading channel model, such as [30,31,32,33,34,35,36,37,38,39,40,41,42] and references therein. By applying the diversity combining schemes, the authors in [34] analyzed the performance of the MRC system over independent and non-identically distributed (i.n.i.d.) Fisher–Snedecor F channels, while the authors in [35] re-investigated the statistical characterization of the sum of i.n.i.d. Fisher–Snedecor F random variables and presented a simple approximation by using another single F random variable. The performance of the PSC scheme with i.n.i.d. branches was analyzed in [36]. The authors in [37] analyzed the channel capacity under different power adaption schemes for MRC diversity system over independent and identically distributed (i.i.d.) F fading channels. The correlated performance of dual-branch MRC and PSC systems over identical Fisher–Snedecor F fading channels was investigated in [38]. Furthermore, the performance of some emerging applications and techniques has also been involved in Fisher–Snedecor F fading channels, such as cascaded fading scenarios [39], spectrum sensing networks [40], dual-hop relaying [41], and free-space optical systems [42].

Although the performance of MRC and PSC systems has been investigated over independent [34,35,36,37] and correlated [38] F fading channels, to the best of the authors’ knowledge, the performance of the SDC systems over independent and correlated Fisher–Snedecor F fading scenarios has yet to be considered in the open research literature. Motivated by the above observation, we will try to fill this gap and provide a comprehensive systematic investigation and certain significant insights about SSC, SEC, and SECps schemes in terms of their potential applications (such as device-to-device, wearable, and Internet of Things applications) over Fisher–Snedecor F fading channels. Hence, the main contributions of this paper are summarized as follows:

(a) We derive an exact and novel analytical expression of the probability density function (PDF) for bivariate Fisher–Snedecor F distribution with arbitrary fading parameters, along with its compact form, using the generalized Lauricella series function.
(b) The analytical expressions of the statistical properties of the output SNR for a dual-branch SSC scheme are deduced in different F scenarios, including independent or correlated and identical or non-identical fading cases. To reduce complicated calculations, only the i.i.d. case is considered for SEC and SECps. Noted that some novel analytical expressions of the moment-generating function (MGF) and the *q*th moments of the output SNR for the SECps scheme are presented in the context of the multivariate Fox’s H-function.
(c) A thorough performance investigation of SSC, SEC, and SECps is presented. The performance metrics of interest comprise the average SNR, amount of fading (AoF), outage probability (OP), average bit error probability and average symbol error probability (ABEP/ASEP), and the average channel capacity. In particular, a novel and exact expression of the ASEP of M-ary quadrature amplitude modulation (MQAM) is also obtained in terms of the multivariate Fox’s H-function. Furthermore, the optimal analysis of the performance metrics in the i.i.d. case is discussed in detail.
(d) Based on the numerical analysis and simulations, certain significant insights are obtained as follows: (i) the impact of the multipath parameter on the system performance is more than those of the other parameters, including the shadowing parameter, the correlation coefficient, and the average SNR; (ii) to explore the potential gains of the SDC systems, the switching threshold should not be too large or too small compared to the average SNR, and there exists an optimal threshold for optimal performance under certain fading scenarios; (iii) SEC and SECps with more than two branches can provide more benefits than SSC when an appropriate switching threshold is chosen. Note that the above new insights will be meaningful and help to enhance the system reliability in the design and deployment of future communications systems.

The remainder of this paper is organized as follows. In Section 2, the statistical properties of the bivariate Fisher–Snedecor F composite distribution are studied. The statistical characteristics of the output SNR of a dual-branch SSC receiver are presented in Section 3, while Section 4 gives the mathematical expressions for the MGF and the moments of the output SNR of multi-branch SEC and SECps in the i.i.d. case. Moreover, Section 5 shows a thorough performance analysis of SSC, SEC, and SECps. Numerical and simulation results are considered and discussed in Section 6. Finally, the main conclusions are summarized in Section 7. The acronyms used across the paper are listed in Table 2.

## 2. Fisher–Snedecor ℱ Fading Channel Model

In this section, we first give the statistical characteristics of the univariate Fisher–Snedecor F distribution. Then, based on these characteristics, the joint PDF of the bivariate Fisher–Snedecor F composite envelope with arbitrary fading parameters is derived. Finally, the joint PDF and the joint cumulative distribution function (CDF) of the instantaneous SNR over the correlated Fisher–Snedecor F composite fading channels are obtained, respectively.

### 2.1. Univariate Fisher–Snedecor F Distribution

The PDF of the instantaneous SNR, *γ*, over the Fisher–Snedecor F composite fading channel is given by [29]:

(1) $$f_{\gamma }(\gamma )=\frac{\Lambda^{m}\gamma^{m-1}}{B(m,n){\left(\Lambda \gamma +1\right)}^{m+n}},$$

where $\Lambda =m/\left(n-1\right)\bar{\gamma }$, which is valid for *n* > 1; *m* and *n* are the multipath fading severity and the shadowing shape parameter, respectively; $\bar{\gamma }=E\left[\gamma \right]E_{s}/N_{0}$ represents the average SNR, where E[⋅] denotes expectation, $E_{s}\;$ is the per symbol energy, and $N_{0}\;$ is the single-sided power spectral density of the complex additive white Gaussian noise (AWGN); B(·,·)  denotes the beta function defined in ([43], Equation (8.384.1)). For the Fisher–Snedecor F composite fading model, the received signals suffer heavy shadowing when  n→1. On the contrary, when n→∞, this indicates the absence of shadowing in wireless fading environments, and this model reduces to a Nakagami-*m* fading channel as a special case. Furthermore, it also comprises Rayleigh (n→∞, m→1) and one-sided Gaussian (n→∞, m→0.5) effects. The corresponding CDF of the instantaneous SNR, *γ* is also given by [29]:

(2) $$F_{\gamma }(\gamma )=\frac{{\left(\Lambda \gamma \right)}^{m}}{mB(m,n)}{}_{2}F_{1}[m+n,m;m+1;-\Lambda \gamma ],$$

where ${}_{2}F_{1}\left[\cdot \right]$ is the Gauss hypergeometric function defined in ([43], Equation (9.100)).

The MGF-based approach is very useful in the performance analysis of wireless digital communication systems. It can be applied to simplify the mathematical analysis and evaluate the ABEP/ASEP and the OP over fading channels. By using (1) and the basic definition of the MGF in [1], and with the aid of ([43], Equation (7.811.5)), the MGF of the instantaneous SNR, *γ* can be derived as:

(3) $$MGF_{\gamma }(\gamma )=\int_{0}^{\infty }\exp(-s\gamma )f_{\gamma }(\gamma )d\gamma =\frac{G_{1,2}^{2,1}[s/\Lambda |{}_{0,n}^{1-m}]}{\Gamma (m)\Gamma (n)},$$

where  Γ (⋅) is the gamma function defined in ([43], Equation (8.310/1)] and $G_{p,q}^{m,n}[\cdot |\cdot ]$ denotes the univariate Meijer G-function defined in ([43], Equation (9.301)). Similarly, the *q*th moment of the instantaneous SNR, *γ* can also be given as:

(4) $$\mu_{\gamma }(q)=\int_{0}^{\infty }\gamma^{q}f_{\gamma }(\gamma )d\gamma =\frac{B(m+q,n-q)}{B(m,n)\Lambda^{q}}.$$

It is noted that (4) is valid only when *n* > *q*.

### 2.2. Bivariate Fisher–Snedecor ℱ Distribution

#### 2.2.1. Joint PDF of Bivariate Fisher–Snedecor ℱ Composite Envelope

Let X*_i_* (*i* = 1, 2) be the channel fading envelopes of Nakagami-𝑚 processes, with the joint PDF between X_1_ and X_2_ given in ([44], Equation (12)) as:

(5) $$\begin{array}{c} f_{X_{1},X_{2}}(x_{1},x_{2})=4{\left(1-\rho_{N}\right)}^{m_{2}}\sum_{k=0}^{\infty }\frac{{\left(m_{1}\right)}_{k}\rho_{N}^{k}}{k!}{}_{1}F_{1}[m_{2}-m_{1},m_{2}+k;\frac{\rho_{N}m_{2}x_{2}^{2}}{Y_{2}(1-\rho_{N})}]\\ \times \prod_{i=1}^{2}{\left[\frac{m_{i}}{Y_{i}(1-\rho_{N})}\right]}^{m_{i}+k}\frac{x_{i}^{2(m_{i}+k)-1}}{\Gamma (m_{i}+k)}\exp\left[-\frac{m_{i}x_{i}^{2}}{(1-\rho_{N})Y_{i}}\right], \end{array}$$

where $m_{2}\geq m_{1}\geq 1/2\;$ is the Nakagami-𝑚 fading parameter, $\rho_{N}$ is the power correlation coefficient between $X_{1}^{2}$, $X_{2}^{2}$ and $Y_{i}$ is the mean fading power $Y_{i}=$ E[$X_{i}^{2}$]. Furthermore, _1_𝐹_1_(⋅;⋅;⋅) is the confluent hypergeometric function defined in ([43], Equation (9.210/1)) and ${\left(x\right)}_{p}=\Gamma \left(x+p\right)/\Gamma \left(x\right)\;$ is the Pochhammer’s symbol with 𝑝 ∈N  defined in ([43], p.xliii).

When multipath fading is superimposed on shadowing, $Y_{i}$ slowly varies and its root mean square can be considered as a random variable following the inverse Nakagami-*m* distribution in [29]. Based on the revised channel model in [45], we let ${\;Y}_{i}=w_{i}^{2}\Omega_{i}\;\left(i=1,2\right)$, where $w_{i}\;$ is a normalized inverse Nakagami-*m* random variable with $\;E\left[w_{i}\right]=1$, $\Omega_{i}=E\left[R_{i}^{2}\right]\;$ is the mean power of the composite signal envelope $R_{i}\;$, then the PDF in (5) is conditioned on ${\;w}_{i}$. To model the inverse Nakagami-*m* distribution, we let the parameter$\;w_{i}=a_{i}/r_{i}$, where $r_{i}$ follows Nakagami-*m* distribution, $a_{i}=\sqrt{(n_{i}-1)/n_{i}}$. Using a standard transformation of random variables, the PDF of the bivariate inverse Nakagami-*m* distribution can be obtained as

(6) $$\begin{array}{c} f_{W_{1},W_{2}}(w_{1},w_{2})=4{\left(1-\rho_{G}\right)}^{n_{2}}\sum_{l=0}^{\infty }\frac{{\left(n_{1}\right)}_{l}\rho_{G}^{l}}{l!}{}_{1}F_{1}[n_{2}-n_{1},n_{2}+l;\frac{\rho_{G}(n_{2}-1)w_{2}^{-2}}{1-\rho_{G}}]\\ \times \prod_{j=1}^{2}{\left[\frac{n_{j}-1}{1-\rho_{G}}\right]}^{n_{j}+l}\frac{w_{j}^{-2(n_{j}+l)-1}}{\Gamma (n_{j}+l)}\exp\left[-\frac{(n_{j}-1)w_{j}^{-2}}{1-\rho_{G}}\right], \end{array}$$

where $n_{2}\geq n_{1}\geq 1/2\;$ is the inverse Nakagami-𝑚 shaping parameter and $\rho_{G}$ is the power correlation coefficient between $w_{1}^{2}$ and $w_{2}^{2}$.

In [29], the PDF of the Fisher–Snedecor F composite envelope is obtained by averaging the conditional PDF of the Nakagami-*m* process over the random variation of the root mean square signal power. To this effect, the PDF of the bivariate Fisher–Snedecor F composite distribution is written as:

(7) $$f_{R_{1},R_{2}}(r_{1},r_{2})=\int_{0}^{\infty }\int_{0}^{\infty }f_{Y_{1}|W_{1},Y_{2}|W_{2}}(r_{1}|w_{1},r_{2}|w_{2})f_{W_{1},W_{2}}(w_{1},w_{2})dw_{1}dw_{2}.$$

Based on (5), $f_{Y_{1}|W_{1},Y_{2}|W_{2}}(r_{1}|w_{1},r_{2}|w_{2})$ in (7) can be obtained as:

(8) $$\begin{array}{c} f_{R_{1}|W_{1},R_{2}|W_{2}}(r_{1}|w_{1},r_{2}|w_{1})=4{\left(1-\rho_{N}\right)}^{m_{2}}\sum_{k=0}^{\infty }\frac{{\left(m_{1}\right)}_{k}\rho_{N}^{k}}{k!}{}_{1}F_{1}[m_{2}-m_{1},m_{2}+k;\frac{\rho_{N}m_{2}r_{2}^{2}}{w_{2}^{2}\Omega_{2}(1-\rho_{N})}]\\ \times \prod_{i=1}^{2}{\left[\frac{m_{i}}{w_{i}^{2}\Omega_{i}(1-\rho_{N})}\right]}^{m_{i}+k}\frac{r_{i}^{2(m_{i}+k)-1}}{\Gamma (m_{i}+k)}\exp\left[-\frac{mr_{i}^{2}}{(1-\rho_{N})w_{i}^{2}\Omega_{i}}\right]. \end{array}$$

Substituting (6) and (8) in (7), and using ([46], Equation (2.2)) with some mathematical manipulation, the joint PDF of the bivariate Fisher–Snedecor F composite distribution with arbitrary fading parameters can be derived as:

(9) $$\begin{array}{ll} f_{R_{1},R_{2}}(r_{1},r_{2}) & =\sum_{k=0}^{\infty }\sum_{l=0}^{\infty }\frac{4{\left(m_{1}\right)}_{k}{\left(n_{1}\right)}_{l}\rho_{N}^{k}\rho_{G}^{l}\Theta }{k!l!B(m_{1}+k,n_{1}+l)B(m_{2}+k,n_{2}+l)}\\ & \times \frac{\beta_{1}^{m_{1}+k}\beta_{2}^{m_{2}+k}r_{1}^{2(m_{1}+k)-1}r_{2}^{2(m_{2}+k)-1}}{{\left(1+\beta_{1}r_{1}^{2}\right)}^{\lambda_{1}}{\left(1+\beta_{2}r_{2}^{2}\right)}^{\lambda_{2}}}\\ & \times F_{2}\left[\lambda_{2};m_{2}-m_{1},n_{2}-n_{1};m_{2}+k,n_{2}+l;\frac{\rho_{N}\beta_{2}r_{2}^{2}}{\beta_{2}r_{2}^{2}+1},\frac{\rho_{G}}{\beta_{2}r_{2}^{2}+1}\right], \end{array}$$

where $\beta_{i}=m_{i}\left(1-\rho_{G}\right)/\left((n_{i}-1\right)\left(1-\rho_{N}\right)\Omega_{i}),$ $\lambda_{i}=m_{i}+k+n_{i}+l$, $\Theta ={\left(1-\rho_{N}\right)}^{m_{2}}{\left(1-\rho_{G}\right)}^{n_{2}}$, (i=1,2), $\;F_{2}\left[\cdot \right]$ is the Appell Hypergeometric function defined in ([43], Equation (9.180.2)). Furthermore, by using the generalized Lauricella series function defined in ([47], Equation (A.20)), the compact form of (9) can be written as:

(10) $$\begin{array}{l} f_{R_{1},R_{2}}(r_{1},r_{2})=\prod_{i=1}^{2}\left(\frac{2\beta_{i}^{m_{i}}r_{i}^{2m_{i}-1}}{{\left(1+\beta_{i}r_{i}^{2}\right)}^{m_{i}+n_{i}}B(m_{i},n_{i})}\right)\\ \times F_{2:0:0:0:0}^{2:0:0:1:1}\left[{}_{\left[(m_{2}:1,0,1,0),(n_{2}:0,1,0,1)]:[-]:[-]:[-]:[-\right]}^{\left[(m_{2}+n_{2}:1,1,1,1),(m_{1}+n_{1}:1,1,0,0)]:[-]:[-]:[(m_{2}-m_{1}:1)]:[(n_{2}-n_{1}:1)\right]}|\rho_{N}A_{1},\rho_{G}A_{2},\rho_{N}A_{3},\rho_{G}A_{4}\right], \end{array}$$

where: $A_{1}=\prod_{i=1}^{2}\frac{\beta_{i}r_{i}^{2}}{\left(1+\beta_{i}r_{i}^{2}\right)},A_{2}=\prod_{i=1}^{2}\frac{1}{\left(1+\beta_{i}r_{i}^{2}\right)},A_{3}=\beta_{2}r_{2}^{2}A_{4},A_{4}=\frac{1}{\left(1+\beta_{2}r_{2}^{2}\right)}$, $F_{2:0:0:0:0}^{2:0:0:1:1}\left[\cdot \right]$ in (10) is defined as:

(11) $$\begin{array}{l} F_{2:0:0:0:0}^{2:0:0:1:1}\left[{}_{\left[(m_{2}:1,0,1,0),(n_{2}:0,1,0,1)]:[-]:[-]:[-]:[-\right]}^{\left[(m_{2}+n_{2}:1,1,1,1),(m_{1}+n_{1}:1,1,0,0)]:[-]:[-]:[(m_{2}-m_{1}:1)]:[(n_{2}-n_{1}:1)\right]}|\rho_{N}A_{1},\rho_{G}A_{2},\rho_{N}A_{3},\rho_{G}A_{4}\right]\\ =\sum_{k=0}^{\infty }\sum_{l=0}^{\infty }\sum_{i=0}^{\infty }\sum_{j=0}^{\infty }\frac{{\left(m_{2}+n_{2}\right)}_{k+l+i+j}{\left(m_{1}+n_{1}\right)}_{k+l}{\left(m_{2}-m_{1}\right)}_{i}{\left(n_{2}-n_{1}\right)}_{j}}{k!l!i!j!{\left(m_{2}\right)}_{k+i}{\left(n_{2}\right)}_{l+j}}\\ \times {\left(\rho_{N}A_{1}\right)}^{k}{\left(\rho_{G}A_{2}\right)}^{l}{\left(\rho_{N}A_{3}\right)}^{i}{\left(\rho_{G}A_{4}\right)}^{j}. \end{array}$$

Additionally, the joint PDF in (10) can be expressed in terms of the multivariable H-function defined in ([47], Equation (A.31)). For the identical shaping parameters case, i.e., $m_{1}=m_{2}=m$ and $n_{1}=n_{2}=n,$ $\;F_{2}\left[\cdot \right]\;$ in (9) equals unity with the aid of Lemma 2.1 in [43] and the identity (9.155.4) defined in [43]. Hence, a simplified formula of (9) can be deduced as ([38], Equation (5)):

(12) $$f_{R_{1},R_{2}}(r_{1},r_{2})=\sum_{k=0}^{\infty }\sum_{l=0}^{\infty }\frac{4\rho_{N}^{k}\rho_{G}^{l}\phi \Gamma (\lambda ){\left(\Xi_{1}\Xi_{2}\right)}^{\left(m+k\right)}}{k!l!\Gamma (m)\Gamma (n)B(k+m,l+n)}\prod_{i=1}^{2}\frac{r_{i}{}^{2m+2k-1}}{{\left(\Xi_{i}r_{i}^{2}+1\right)}^{\lambda }}$$

where $\Xi_{i}=m\left(1-\rho_{G}\right)/\left(\left(n-1\right)\left(1-\rho_{N}\right)\Omega_{i}\right)$, λ=m+k+n+l, $\phi ={\left(1-\rho_{N}\right)}^{m}{\left(1-\rho_{G}\right)}^{n}$.

#### 2.2.2. Joint PDF of the Instantaneous Output SNR

Let $\;\gamma_{i}=R_{i}^{2}E_{s}/N_{0}\;\left(i=1,\;2\right)\;$ denote the instantaneous output SNR of the bivariate Fisher–Snedecor F composite fading model. By using (9) and a simple variable transformation, the joint PDF of $\gamma_{1}$ and $\gamma_{2}\;$ for the bivariate Fisher–Snedecor F composite fading model can be expressed as follows:

(13) $$\begin{array}{ll} f_{\gamma_{1},\gamma_{2}}(\gamma_{1},\gamma_{2})= & \sum_{k=0}^{\infty }\sum_{l=0}^{\infty }\frac{{\left(m_{1}\right)}_{k}\rho_{N}^{k}{\left(n_{1}\right)}_{l}\rho_{G}^{l}\Theta \alpha_{1}^{m_{1}+k}\alpha_{2}^{m_{2}+k}}{k!l!B(m_{1}+k,n_{1}+l)B(m_{2}+k,n_{2}+l)}\frac{\gamma_{1}^{(m_{1}+k)-1}\gamma_{2}^{(m_{2}+k)-1}}{{\left(1+\alpha_{1}\gamma_{1}\right)}^{\lambda_{1}}{\left(1+\alpha_{2}\gamma_{2}\right)}^{\lambda_{2}}}\\ & \times F_{2}\left[\lambda_{2};m_{2}-m_{1},n_{2}-n_{1};m_{2}+k,n_{2}+l;\frac{\rho_{N}\alpha_{2}\gamma_{2}}{\alpha_{2}\gamma_{2}+1},\frac{\rho_{G}}{\alpha_{2}\gamma_{2}+1}\right], \end{array}$$

where $\alpha_{i}=m_{i}\left(1-\rho_{G}\right)/\left((n_{i}-1)\left(1-\rho_{N}\right){\bar{\gamma }}_{i}\right),$ ${\bar{\gamma }}_{i}=\Omega_{i}E_{s}/N_{0}.\;$ By using (12) and the same approach as (13), the joint PDF of $\gamma_{1}$ and $\gamma_{2}$ for the special case of identical shaping parameters can be obtained as:

(14) $$\begin{array}{ll} f_{\gamma_{1},\gamma_{2}}(\gamma_{1},\gamma_{2}) & =\frac{\phi }{B^{2}(m,n)}\left(\prod_{i=1}^{2}\frac{\eta_{i}{}^{m}\gamma_{i}^{m-1}}{{\left(\eta_{i}\gamma_{i}+1\right)}^{m+n}}\right)\\ & \times F_{4}\left[m+n,m+n;m,n;\prod_{i=1}^{2}\frac{\rho_{N}\eta_{i}\gamma_{i}}{\eta_{i}\gamma_{i}+1},\prod_{i=1}^{2}\frac{\rho_{G}}{\eta_{i}\gamma_{i}+1}\right]. \end{array}$$

where $\eta_{i}=m\left(1-\rho_{G}\right)/\left(\left(n-1\right)\left(1-\rho_{N}\right){\bar{\gamma }}_{i}\right)$, and $\;F_{4}\left[\cdot \right]$ is the Appell hypergeometric function defined in ([43], Equation (9.180.4)).

#### 2.2.3. Joint CDF of the Instantaneous Output SNR

To obtain an analytical expression of the joint CDF of the instantaneous output SNR in the bivariate Fisher–Snedecor F model, we express Appell’s function in (13) as the infinite series representations using ([43], Equation (9.180.2)). With the help of ([43], Equation (3.194.1)), along with some algebraic manipulations, the joint CDF of $\gamma_{1}$ and $\gamma_{2}$ can be deduced as:

(15) $$\begin{array}{ll} F_{\gamma_{1},\gamma_{2}}(\gamma_{1},\gamma_{2}) & =\int_{0}^{\gamma_{1}}\int_{0}^{\gamma_{2}}f_{\gamma_{1},\gamma_{2}}(\gamma_{1},\gamma_{2})d\gamma_{1}d\gamma_{2}\\ & =\sum_{k=0}^{\infty }\sum_{l=0}^{\infty }\sum_{i=0}^{\infty }\sum_{j=0}^{\infty }\frac{\rho_{N}^{k+i}\rho_{G}^{l+j}\Theta \alpha_{1}^{m_{1}+k}\alpha_{2}^{m_{2}+k+i}\gamma_{1}^{m_{1}+k}\gamma_{2}^{m_{2}+k+i}}{k!l!i!j!\Gamma (m_{1})\Gamma (n_{1})\Gamma (m_{2}+k+i)}\\ & \times \frac{{\left(m_{2}-m_{1}\right)}_{i}{\left(n_{2}-n_{1}\right)}_{j}\Gamma (\lambda_{1})\Gamma (\lambda_{2}+i+j)}{\Gamma (n_{2}+l+j)(m_{1}+k)(m_{2}+k+i)}\\ & \times {}_{2}F_{1}[\lambda_{1},m_{1}+k;m_{1}+k+1;-\alpha_{1}\gamma_{1}]\\ & \times {}_{2}F_{1}[\lambda_{2}+i+j,m_{2}+k+i;m_{2}+k+i+1;-\alpha_{2}\gamma_{2}]. \end{array}$$

Similarly, with the aid of the infinite series representations of $F_{4}\left[\cdot \right]$ in [46], and invoking ([43], Equation (9.180.2)) and ([43], Equation (3.194.1/3)), the joint CDF for the identical shaping parameters case can be obtained as ([38], Equation (19)):

(16) $$\begin{array}{ll} F_{\gamma_{1},\gamma_{2}}(\gamma_{1},\gamma_{2})= & \sum_{k=0}^{\infty }\sum_{l=0}^{\infty }\frac{\rho_{N}^{k}\rho_{G}^{l}\phi {\left(\eta_{1}\eta_{2}\right)}^{m+k}\Gamma (\lambda ){\left(m+k\right)}^{-2}}{k!l!\Gamma (m)\Gamma (n)B(l+n,k+m)}\\ & \times \prod_{i=1}^{2}\gamma_{i}{}^{m+k}{}_{2}F_{1}[\lambda ,m+k;1+m+k,-\eta_{i}\gamma_{i}]. \end{array}$$

## 3. Dual-Branch SSC Receiver

In this section, we consider a dual-branch SSC diversity receiver operating over both non-independent (correlated) and non-identically distributed (n.i.n.i.d.) Fisher–Snedecor F composite fading channels. The equivalent baseband received signal at the *i*th (*i* = 1, 2) antenna can be expressed as $r_{i}=sh_{i}+n_{i}$, where *s* is the transmitted complex symbol with energy $E_{s}=$ E[${\left|s\right|}^{2}$], $n_{i}$ is the complex AWGN with $N_{0}$ assumed to be identical and uncorrelated to both branches, and $h_{i}$ is the complex channel gain with its magnitude $R_{i}=\left|h_{i}\right|$ being modeled as a Fisher–Snedecor F distributed random variable. Furthermore, the general assumptions are made that only the channel fading magnitude affects the received signal and that the phase can be accurately estimated.

Let $\gamma_{SCC}$ represent the instantaneous SNR per symbol at the output of the SSC and $\;\gamma_{\tau }$ denote the predetermined switching threshold. In the non-identical distribution scenarios, two branches of the SSC can have different average SNRs or different fading parameters in the same fading distributions, even if they follow different fading distributions.

### 3.1. PDF

According to Equation (4) in [5], the PDF of the output SNR of the SSC over n.i.n.i.d. Fisher–Snedecor composite fading channels can be given by:

(17) $$f_{\gamma_{ssc}}(\gamma )=\{\begin{array}{ll} P\sum_{i=1}^{2}\left(g_{\gamma_{i}}(\gamma )/F_{i}\right), & \gamma \leq \gamma_{\tau };\\ P\sum_{i=1}^{2}\left[\left(g_{\gamma_{i}}(\gamma )+f_{\gamma_{i}}(\gamma )\right)/F_{i}\right], & \gamma >\gamma_{\tau }, \end{array}$$

where $P=F_{1}F_{2}/\left(F_{1}+F_{2}\right)$, $F_{i}=F_{\gamma_{i}}\left(\gamma_{\tau }\right)$ (*i* = 1, 2) can be obtained by using (2), $f_{\gamma_{i}}\left(\gamma \right)\;$ can be obtained by using (1), and $g_{\gamma_{1}}\left(\gamma \right)$ and $g_{\gamma_{2}}\left(\gamma \right)$ are defined as:

(18a) $$g_{\gamma_{1}}(\gamma )=\int_{0}^{\gamma_{\tau }}f_{\gamma_{1},\gamma_{2}}(\gamma_{1},\gamma )d\gamma_{1},$$

(18b) $$g_{\gamma_{2}}(\gamma )=\int_{0}^{\gamma_{\tau }}f_{\gamma_{1},\gamma_{2}}(\gamma ,\gamma_{2})d\gamma_{2}.$$

We substitute (13) into (18a) and use the infinite series representations of the Appell’s function defined in ([43], Equation (9.180.2)), and with the help of ([43], Equation (3.194.1)) along with some mathematical manipulation, $g_{\gamma_{1}}\left(\gamma \right)\;$ can be solved as:

(19) $$\begin{array}{ll} g_{\gamma_{1}}(\gamma )= & \sum_{k=0}^{\infty }\sum_{l=0}^{\infty }\sum_{i=0}^{\infty }\sum_{j=0}^{\infty }\frac{\rho_{N}^{k+i}\rho_{G}^{l+j}\Theta \Gamma (\lambda_{1}){\left(m_{2}-m_{1}\right)}_{i}{\left(n_{2}-n_{1}\right)}_{j}\alpha_{1}^{m_{1}+k}\alpha_{2}^{m_{2}+k+i}\gamma_{\tau }^{m_{1}+k}}{k!l!i!j!\Gamma (m_{1})\Gamma (n_{1})B(m_{2}+k+i,n_{2}+l+j)(m_{1}+k)}\\ & \times \gamma^{m_{2}+k+i-1}{\left(1+\alpha_{2}\gamma \right)}^{-(\lambda_{2}+i+j)}{}_{2}F_{1}[\lambda_{1},m_{1}+k;1+m_{1}+k,-\alpha_{1}\gamma_{\tau }]. \end{array}$$

Likewise, by substituting (13) into (18b), $g_{\gamma_{2}}\left(\gamma \right)$ can be solved as:

(20) $$\begin{array}{ll} g_{\gamma_{2}}(\gamma )= & \sum_{k=0}^{\infty }\sum_{l=0}^{\infty }\sum_{i=0}^{\infty }\sum_{j=0}^{\infty }\frac{\rho_{N}^{k+i}\rho_{G}^{l+j}\Theta \Gamma (\lambda_{1}){\left(m_{2}-m_{1}\right)}_{i}{\left(n_{2}-n_{1}\right)}_{j}\alpha_{1}^{m_{1}+k}\alpha_{2}^{m_{2}+k+i}\gamma_{\tau }^{m_{2}+k+i}}{k!l!i!j!\Gamma (m_{1})\Gamma (n_{1})B(m_{2}+k+i,n_{2}+l+j)(m_{2}+k+i)}\\ & \times \gamma^{m_{1}+k-1}{\left(1+\alpha_{1}\gamma \right)}^{-\lambda_{1}}{}_{2}F_{1}[\lambda_{2}+i+j,m_{2}+k+i;m_{2}+k+i+1;-\alpha_{2}\gamma_{\tau }]. \end{array}$$

For the non-independent and identically distributed (n.i.i.d.) Fisher–Snedecor F composite fading scenario, $f_{\gamma_{1}}\left(\cdot \right)=f_{\gamma_{2}}\left(\cdot \right)$, $F_{\gamma_{1}}\left(\cdot \right)=F_{\gamma_{2}}\left(\cdot \right)$, and $g_{\gamma_{1}}\left(\cdot \right)=g_{\gamma_{2}}\left(\cdot \right)$, Equation (17) can be rewritten as:

(21) $$f_{\gamma_{ssc}}(\gamma )=\{\begin{array}{ll} g_{\gamma_{2}}(\gamma ), & \gamma \leq \gamma_{\tau };\\ g_{\gamma_{2}}(\gamma )+f_{\gamma_{2}}(\gamma ), & \gamma >\gamma_{\tau }. \end{array}$$

By substituting (14) into (18b) with the help of the infinite series representations of $\;F_{4}\left[\cdot \right]\;$ in ([43], Equation (9.180.4)) and ([43], Equation (3.194.3)), after some mathematical manipulation, $g_{\gamma_{2}}\left(\gamma \right)$ can be solved as:

(22) $$\begin{array}{ll} g_{\gamma_{2}}(\gamma )= & \sum_{k=0}^{\infty }\sum_{l=0}^{\infty }\frac{\rho_{N}^{k}\rho_{G}^{l}\phi \Gamma (\lambda ){\left(\eta_{1}\eta_{2}\right)}^{\left(m+k\right)}\gamma_{\tau }^{m+k}\gamma^{m+k-1}}{l!k!\Gamma (m)\Gamma (n)B(k+m,l+n)(m+k)}\\ & \times {\left(\eta_{1}\gamma +1\right)}^{-\lambda }{}_{2}F_{1}[\lambda ,m+k;1+m+k,-\eta_{2}\gamma_{\tau }]. \end{array}$$

While for the i.n.i.d. case, $g_{\gamma_{1}}\left(\gamma \right)=F_{\gamma_{1}}\left(\gamma_{\tau }\right)\cdot f_{\gamma_{2}}\left(\gamma \right)$ and $g_{\gamma_{2}}\left(\gamma \right)=F_{\gamma_{2}}\left(\gamma_{\tau }\right)\cdot f_{\gamma_{1}}\left(\gamma \right)$, Equation (17) can be rewritten as:

(23) $$f_{\gamma_{ssc}}(\gamma )=\{\begin{array}{ll} P\sum_{i=1}^{2}f_{\gamma_{i}}(\gamma ), & \gamma \leq \gamma_{\tau };\\ P\sum_{i=1}^{2}f_{\gamma_{i}}(\gamma )(1+1/F_{i}), & \gamma >\gamma_{\tau }. \end{array}$$

When the i.i.d. case is considered, Equation (23) can be simplified as:

(24) $$f_{\gamma_{ssc}}(\gamma )=\{\begin{array}{ll} F_{\gamma }(\gamma_{\tau })f_{\gamma }(\gamma ), & \gamma \leq \gamma_{\tau };\\ {}[1+F_{\gamma }(\gamma_{\tau })]f_{\gamma }(\gamma ), & \gamma >\gamma_{\tau }. \end{array}$$

where $F_{\gamma }\left(\gamma_{\tau }\right)$ and $f_{\gamma }\left(\gamma \right)$ can be obtained using (1) and (2), respectively.

### 3.2. CDF

By integrating the expression of $f_{\gamma_{SCC}}\left(\gamma \right)$ in (17) with respect to  γ, the corresponding CDF of $\gamma_{SCC}$ over n.i.n.i.d. Fisher–Snedecor fading channels can be expressed as:

(25) $$F_{\gamma_{ssc}}(\gamma )=\{\begin{array}{ll} PS_{1}, & \gamma \leq \gamma_{\tau };\\ P(S_{1}+S_{2}), & \gamma >\gamma_{\tau }, \end{array}$$

where $S_{1}=F_{\gamma_{1},\gamma_{2}}\left(\gamma_{\tau },\gamma \right)/F_{1}+F_{\gamma_{1},\gamma_{2}}\left(\gamma ,\gamma_{\tau }\right)/F_{2}$, $S_{2}=\left[F_{\gamma_{1}}\left(\gamma \right)-F_{\gamma_{1}}\left(\gamma_{\tau }\right)\right]/F_{1}+\left[F_{\gamma_{2}}\left(\gamma \right)-F_{\gamma_{2}}\left(\gamma_{\tau }\right)\right]/F_{2}$, $F_{\gamma_{i}}\left(\gamma \right)$ and $\;F_{\gamma_{i}}\left(\gamma_{\tau }\right)$ can be obtained by using (2). $F_{\gamma_{1},\gamma_{2}}\left(\gamma_{\tau },\gamma \right)$ and $F_{\gamma_{1},\gamma_{2}}\left(\gamma ,\gamma_{\tau }\right)\;$ can be yielded by using (15) and replacing $\;\left(\gamma_{1},\gamma_{2}\right)$ with $\left(\gamma_{\tau },\gamma \right)$ and $\left(\gamma ,\gamma_{\tau }\right)$, respectively.

Similarly, under the n.i.i.d. case, the CDF in (25) can be expressed as:

(26) $$F_{\gamma_{ssc}}(\gamma )=\{\begin{array}{ll} F_{\gamma_{1},\gamma_{2}}(\gamma ,\gamma_{\tau }), & \gamma \leq \gamma_{\tau };\\ F_{\gamma_{2}}(\gamma )-F_{\gamma_{2}}(\gamma_{\tau })+F_{\gamma_{1},\gamma_{2}}(\gamma ,\gamma_{\tau }), & \gamma >\gamma_{\tau }. \end{array}$$

While for the i.n.i.d. case, the CDF in (25) can be reduced as:

(27) $$F_{\gamma_{ssc}}(\gamma )=\{\begin{array}{ll} P\sum_{i=1}^{2}F_{\gamma_{i}}(\gamma ), & \gamma \leq \gamma_{\tau };\\ P\sum_{i=1}^{2}\left[F_{\gamma_{i}}(\gamma )(1+1/F_{i})-1\right], & \gamma >\gamma_{\tau }. \end{array}$$

Finally, for the i.i.d. case, the CDF in (27) can be further simplified as:

(28) $$F_{\gamma_{ssc}}(\gamma )=\{\begin{array}{ll} F_{\gamma }(\gamma_{\tau })F_{\gamma }(\gamma ), & \gamma \leq \gamma_{\tau };\\ F_{\gamma }(\gamma )-F_{\gamma }(\gamma_{\tau })+F_{\gamma }(\gamma_{\tau })F_{\gamma }(\gamma ), & \gamma >\gamma_{\tau }. \end{array}$$

### 3.3. MGF

Based on the definition of MGF and using (17), the MGF of the output SNR of the SSC over n.i.n.i.d. Fisher–Snedecor F composite fading channels can be written as:

(29) $$MGF_{\gamma_{ssc}}(s)=\sum_{i=1}^{2}\left(P/F_{i})MGF_{\gamma_{ssc}}^{\left(\gamma_{i}\right)}(s\right),$$

where:

(30) $$MGF_{\gamma_{ssc}}^{\left(\gamma_{i}\right)}(s)=\int_{0}^{\infty }\exp(-s\gamma )g_{\gamma_{i}}(\gamma )d\gamma +\int_{\gamma_{\tau }}^{\infty }\exp(-s\gamma )f_{\gamma_{i}}(\gamma )d\gamma .$$

By applying (1), (19) and (20), and after some mathematical manipulation, $MGF_{\gamma_{SSC}}^{\left(\gamma_{1}\right)}\left(s\right)$ and $MGF_{\gamma_{SSC}}^{\left(\gamma_{2}\right)}\left(s\right)$ are derived, respectively, as (see Appendix A for details):

(31a) $$\begin{array}{ll} MGF_{\gamma_{ssc}}^{\left(\gamma_{1}\right)}(s) & =\sum_{k=0}^{\infty }\sum_{l=0}^{\infty }\sum_{i=0}^{\infty }\sum_{j=0}^{\infty }\frac{\rho_{N}^{k+i}\rho_{G}^{l+j}\Theta \Gamma (\lambda_{1}){\left(m_{2}-m_{1}\right)}_{i}{\left(n_{2}-n_{1}\right)}_{j}\alpha_{1}^{m_{1}+k}\gamma_{\tau }^{m_{1}+k}}{k!l!i!j!\Gamma (m_{1})\Gamma (n_{1})\Gamma (m_{2}+k+i)\Gamma (n_{2}+l+j)(m_{1}+k)}\\ & \times G_{1,2}^{2,1}[s/\alpha_{2}|{}_{0,n_{2}+l+j}^{1-(m_{2}+k+i)}]{}_{2}F_{1}[\lambda_{1},m_{1}+k;1+m_{1}+k,-\alpha_{1}\gamma_{\tau }]\\ & +\frac{G_{1,2}^{2,1}[s/\Lambda_{1}|{}_{0,n_{1}}^{1-m_{1}}]}{\Gamma (m_{1})\Gamma (n_{1})}-\frac{\Lambda_{1}^{m_{1}}\gamma_{\tau }^{m_{1}}}{\Gamma (m_{1})\Gamma (n_{1})}G_{1,1:0,1:1,1}^{0,1:1,0:1,1}[s\gamma_{\tau },\gamma_{\tau }\Lambda_{1}|{}_{-m_{1}}^{1-m_{1}}|{}_{0}^{-}|{}_{0}^{1-(m_{1}+n_{1})}], \end{array}$$

(31b) $$\begin{array}{cc} MGF_{\gamma_{ssc}}^{\left(\gamma_{2}\right)}(s) & =\sum_{k=0}^{\infty }\sum_{l=0}^{\infty }\sum_{i=0}^{\infty }\sum_{j=0}^{\infty }\frac{\rho_{N}^{k+i}\rho_{G}^{l+j}\Theta {\left(m_{2}-m_{1}\right)}_{i}{\left(n_{2}-n_{1}\right)}_{j}\alpha_{2}^{m_{2}+k+i}\gamma_{\tau }^{m_{2}+k+i}}{k!l!i!j!\Gamma (m_{1})\Gamma (n_{1})B(m_{2}+k+i,n_{2}+l+j)(m_{2}+k+i)}\\ & \times G_{1,2}^{2,1}[s/\alpha_{1}|{}_{0,n_{1}+l}^{1-(m_{1}+k)}]{}_{2}F_{1}[\lambda_{2}+i+j,m_{2}+k+i;m_{2}+k+i+1;-\alpha_{2}\gamma_{\tau }]\\ & +\frac{G_{1,2}^{2,1}[s/\Lambda_{2}|{}_{0,n_{2}}^{1-m_{2}}]}{\Gamma (m_{2})\Gamma (n_{2})}-\frac{\Lambda_{2}^{m_{2}}\gamma_{\tau }^{m_{2}}}{\Gamma (m_{2})\Gamma (n_{2})}G_{1,1:0,1:1,1}^{0,1:1,0:1,1}[s\gamma_{\tau },\gamma_{\tau }\Lambda_{2}|{}_{-m_{2}}^{1-m_{2}}|{}_{0}^{-}|{}_{0}^{1-(m_{2}+n_{2})}], \end{array}$$

where $G_{p,q:p_{1},q_{1}:p_{2},q_{2}}^{m,n:m_{1},n_{1}:m_{2},n_{2}}\left[\cdot \right]$ denotes a bivariate Meijer G-function defined in ([48], Equation (13.1)). For the n.i.i.d. case, the MGF in (29) can be simplified as:

(32) $$MGF_{\gamma_{ssc}}(s)=\int_{0}^{\infty }\exp(-s\gamma )g_{\gamma_{2}}(\gamma )d\gamma +\int_{\gamma_{\tau }}^{\infty }\exp(-s\gamma )f_{\gamma_{2}}(\gamma )d\gamma .$$

By substituting (1) and (22) into (32) with the aid of ([43], Equation (3.194.3)), and performing the same method as Appendix A, the analytical expression of ${\mathrm{MGF}}_{\gamma_{SCC}}\left(s\right)$ over n.i.i.d. Fisher–Snedecor F composite fading channels can be given as follows:

(33) $$\begin{array}{ll} MGF_{\gamma_{ssc}}(s) & =\sum_{k=0}^{\infty }\sum_{l=0}^{\infty }\frac{\rho_{N}^{k}\rho_{G}^{l}\phi G_{1,2}^{2,1}[s/\eta_{1}|{}_{0,n+l}^{1-(m+k)}]}{l!k!\Gamma (m)\Gamma (n)B(k+m,l+n)(m+k)}\\ & \times {\left(\gamma_{\tau }\eta_{2}\right)}^{m+k}{}_{2}F_{1}[\lambda ,m+k;1+m+k,-\eta_{2}\gamma_{\tau }]\\ & +\frac{G_{1,2}^{2,1}[s/\Lambda_{2}|{}_{0,n}^{1-m}]}{\Gamma (m)\Gamma (n)}-\frac{\Lambda_{2}^{m}\gamma_{\tau }^{m}}{\Gamma (m)\Gamma (n)}G_{1,1:0,1:1,1}^{0,1:1,0:1,1}[s\gamma_{\tau },\gamma_{\tau }\Lambda_{2}|{}_{-m}^{1-m}|{}_{0}^{-}|{}_{0}^{1-(m+n)}]. \end{array}$$

While for the i.n.i.d. case, the MGF in (29) can be simplified as:

(34) $$MGF_{\gamma_{ssc}}(s)=P\sum_{i=1}^{2}\left(\underset{I_{1}}{\underbrace{\int_{0}^{\infty }\exp(-s\gamma )f_{\gamma_{i}}(\gamma )d\gamma }}+\underset{I_{2}}{\underbrace{F_{i}^{-1}\int_{\gamma_{\tau }}^{\infty }\exp(-s\gamma )f_{\gamma_{i}}(\gamma )d\gamma }}\right),$$

where the first integral *I*_1_ in (34) can be obtained as (3), the second integral *I*_2_ in (34) can be derived as (A3), then the closed-form expression of the MGF in (34) can be obtained as:

(35) $$MGF_{\gamma_{ssc}}(s)=P\sum_{i=1}^{2}\left(\frac{\left(1+F_{i}^{-1})G_{1,2}^{2,1}[s/\Lambda_{i}|{}_{0,n_{i}}^{1-m_{i}}\right]}{\Gamma (m_{i})\Gamma (n_{i})}-\frac{F_{i}^{-1}\Lambda_{i}^{m_{i}}\gamma_{\tau }^{m_{i}}}{\Gamma (m_{i})\Gamma (n_{i})}G_{1,1:0,1:1,1}^{0,1:1,0:1,1}[s\gamma_{\tau },\gamma_{\tau }\Lambda_{i}|{}_{-m_{i}}^{1-m_{i}}|{}_{0}^{-}|{}_{0}^{1-(m_{i}+n_{i})}]\right).$$

Similarly, on the basis of (34), the MGF of the output SNR of the SSC for the i.i.d. case can be further obtained as:

(36) $$MGF_{\gamma_{ssc}}(s)=F_{\gamma }(\gamma_{\tau })\int_{0}^{\infty }\exp(-s\gamma )f_{\gamma }(\gamma )d\gamma +\int_{\gamma_{\tau }}^{\infty }\exp(-s\gamma )f_{\gamma }(\gamma )d\gamma .$$

Hence, the closed-form expression of the MGF in (36) can also be readily obtained as:

(37) $$MGF_{\gamma_{ssc}}(s)=\frac{\left(1+F_{\gamma }(\gamma_{\tau }))G_{1,2}^{2,1}[s/\Lambda |{}_{0,n}^{1-m}\right]}{\Gamma (m)\Gamma (n)}-\frac{\Lambda^{m}\gamma_{\tau }^{m}}{\Gamma (m)\Gamma (n)}G_{1,1:0,1:1,1}^{0,1:1,0:1,1}[s\gamma_{\tau },\gamma_{\tau }\Lambda |{}_{-m}^{1-m}|{}_{0}^{-}|{}_{0}^{1-(m+n)}].$$

### 3.4. The qth Moments

Based on the definition of the *q*th moments and using (17), $\mu_{\gamma_{SCC}}\left(q\right)$ in the n.i.n.i.d. case can be expressed as:

(38) $$\mu_{\gamma_{ssc}}(q)=\sum_{i=1}^{2}\left(P/F_{i}\right)\mu_{\gamma_{ssc}}^{\left(\gamma_{i}\right)}(q),$$

where $\mu_{\gamma_{SSC}}^{\left(\gamma_{i}\right)}\left(q\right)$ is defined as:

(39) $$\mu_{\gamma_{ssc}}^{\left(\gamma_{i}\right)}(q)=\int_{0}^{\infty }\gamma^{q}g_{\gamma_{i}}(\gamma )d\gamma +\int_{\gamma_{\tau }}^{\infty }\gamma^{q}f_{\gamma_{i}}(\gamma )d\gamma .$$

By substituting $g_{\gamma_{1}}\left(\gamma \right)$ in (19) (or $g_{\gamma_{2}}\left(\gamma \right)\;$ in (20)) and $f_{\gamma_{1}}\left(\gamma \right)\;$ (or $\;f_{\gamma_{2}}\left(\gamma \right)$) in (1) and using ([43], Equation (3.194.2/3)), after some mathematical manipulation, $\mu_{\gamma_{SSC}}^{\left(\gamma_{1}\right)}\left(q\right)$ and $\mu_{\gamma_{SSC}}^{\left(\gamma_{2}\right)}\left(q\right)\;$ are respectively yielded as:

(40) $$\begin{array}{ll} \mu_{\gamma_{ssc}}^{\left(\gamma_{1}\right)}(q) & =\sum_{k=0}^{\infty }\sum_{l=0}^{\infty }\sum_{i=0}^{\infty }\sum_{j=0}^{\infty }\frac{\rho_{N}^{k+i}\rho_{G}^{l+j}\Theta \Gamma (\lambda_{1}){\left(m_{2}-m_{1}\right)}_{i}{\left(n_{2}-n_{1}\right)}_{j}\alpha_{1}^{m_{1}+k}\alpha_{2}^{-q}\gamma_{\tau }^{m_{1}+k}}{k!l!i!j!\Gamma (m_{1})\Gamma (n_{1})B(m_{2}+k+i,n_{2}+l+j)(m_{1}+k)}\\ & \times B(m_{2}+k+i+q,n_{2}+l+j-q){}_{2}F_{1}[\lambda_{1},m_{1}+k;1+m_{1}+k,-\alpha_{1}\gamma_{\tau }]\\ & +\frac{\gamma_{\tau }^{q-n_{1}}\Lambda_{1}^{-n_{1}}}{B(m_{1},n_{1})(n_{1}-q)}{}_{2}F_{1}[m_{1}+n_{1},n_{1}-q;n_{1}-q+1;-{\left(\Lambda_{1}\gamma_{\tau }\right)}^{-1}], \end{array}$$

(41) $$\begin{array}{ll} \mu_{\gamma_{ssc}}^{\left(\gamma_{2}\right)}(q) & =\sum_{k=0}^{\infty }\sum_{l=0}^{\infty }\sum_{i=0}^{\infty }\sum_{j=0}^{\infty }\frac{\rho_{N}^{k+i}\rho_{G}^{l+j}\Theta \Gamma (\lambda_{1}){\left(m_{2}-m_{1}\right)}_{i}{\left(n_{2}-n_{1}\right)}_{j}\alpha_{1}^{-q}\alpha_{2}^{m_{2}+k+i}\gamma_{\tau }^{m_{2}+k+i}}{k!l!i!j!\Gamma (m_{1})\Gamma (n_{1})B(m_{2}+k+i,n_{2}+l+j)(m_{2}+k+i)}\\ & \times B(m_{1}+k+q,n_{1}+l-q){}_{2}F_{1}[\lambda_{2}+i+j,m_{2}+k+i;m_{2}+k+i+1;-\alpha_{2}\gamma \\ & +\frac{\gamma_{\tau }^{q-n_{2}}\Lambda_{2}^{-n_{2}}}{B(m_{2},n_{2})(n_{2}-q)}{}_{2}F_{1}[m_{2}+n_{2},n_{2}-q;n_{2}-q+1;-{\left(\Lambda_{2}\gamma_{\tau }\right)}^{-1}]. \end{array}$$

In the case of n.i.i.d. Fisher–Snedecor composite fading channels, the *q*th order moment of $\gamma_{SCC}\;$ can be expressed as:

(42) $$\mu_{\gamma_{ssc}}(q)=\int_{0}^{\infty }\gamma^{q}g_{\gamma_{2}}(\gamma )d\gamma +\int_{\gamma_{\tau }}^{\infty }\gamma^{q}f_{\gamma_{2}}(\gamma )d\gamma .$$

By substituting (1) and (22) into (41) with the aid of ([43], Equation (3.194.3)), $\mu_{\gamma_{SCC}}\left(q\right)$ can be obtained as:

(43) $$\begin{array}{ll} \mu_{\gamma_{ssc}}(q) & =\sum_{k=0}^{\infty }\sum_{l=0}^{\infty }\frac{\rho_{N}^{k}\rho_{G}^{l}\Gamma (\lambda )B(m+k+q,n+l-q)}{k!l!\Gamma (m)\Gamma (n)B(l+n,k+m)}\\ & \times \frac{\phi {\left(\gamma_{\tau }\eta_{2}\right)}^{m+k}}{\eta_{1}^{q}(m+k)}{}_{2}F_{1}[\lambda ,m+k;m+k+1;-\eta_{2}\gamma_{\tau }]\\ & +\frac{\gamma_{\tau }^{q-n}\Lambda_{2}^{-n}}{B(m,n)(n-q)}{}_{2}F_{1}[m+n,n-q;n-q+1;-1/\Lambda_{2}\gamma_{\tau }]. \end{array}$$

For the i.n.i.d. case, the *q*th order moment of $\gamma_{SCC}$ in (38) can be simplified as:

(44) $$\mu_{\gamma_{ssc}}(q)=P\sum_{i=1}^{2}\left(\int_{0}^{\infty }\gamma^{q}f_{\gamma_{i}}(\gamma )d\gamma +F_{i}^{-1}\int_{\gamma_{\tau }}^{\infty }\gamma^{q}f_{\gamma_{i}}(\gamma )d\gamma \right).$$

By using (12) and ([43], Equation (3.194.2/3)) along with some mathematical manipulation, the closed-form expression of (44) can be calculated. Similarly, we also obtain the closed-form expression of the *q*th order moment of $\gamma_{SCC}$ for the i.i.d. case.

## 4. Multi-Branch SEC Receiver

In this section, we consider an *L*-branch SEC receiver and an *L*-branch SECps receiver over i.i.d. Fisher–Snedecor composite fading channels. Let $\gamma_{SEC}$ and $\gamma_{SECps}$ represent the instantaneous SNR per symbol at the output of the SEC and SECps, respectively. These systems always use one predetermined switching threshold, $\gamma_{\tau }$.

### 4.1. SEC Receiver

The PDF of the instantaneous SNR, $\;\gamma_{SEC}$, at the SEC output can be expressed as [3]:

(45) $$f_{\gamma_{SEC}}(\gamma )=\{\begin{array}{cc} P_{1}f_{\gamma }(\gamma ), & \gamma \leq \gamma_{\tau };\\ P_{2}f_{\gamma }(\gamma ), & \gamma >\gamma_{\tau }, \end{array}$$

where $P_{1}={\left[F_{\gamma }\left(\gamma_{\tau }\right)\right]}^{L-1}$, $\;P_{2}=(1-{\left[F_{\gamma }\left(\gamma_{\tau }\right)\right]}^{L})/\left(1-F_{\gamma }\left(\gamma_{\tau }\right)\right)$. $f_{\gamma }\left(\gamma \right)\;$ and $F_{\gamma }\left(\gamma_{\tau }\right)$ can be obtained by using (1) and (2), respectively. By integrating the expression of $f_{\gamma_{SEC}}\left(\gamma \right)$ in (45) with respect to γ, the corresponding CDF of $\gamma_{SEC}$ can be written as:

(46) $$F_{\gamma_{SEC}}(\gamma )=\{\begin{array}{cc} P_{1}F_{\gamma }(\gamma ), & \gamma \leq \gamma_{\tau };\\ P_{2}F_{\gamma }(\gamma ), & \gamma >\gamma_{\tau }. \end{array}$$

Similar to (41), the *q*th moments of $\gamma_{SEC}$ can be given as:

(47) $$\mu_{\gamma_{SEC}}(q)=P_{1}\int_{0}^{\infty }\gamma^{q}f_{\gamma }(\gamma )d\gamma +P_{3}\int_{\gamma_{\tau }}^{\infty }\gamma^{q}f_{\gamma }(\gamma )d\gamma ,$$

where $P_{3}=(1-{\left[F_{\gamma }\left(\gamma_{\tau }\right)\right]}^{L-1})/\left(1-F_{\gamma }\left(\gamma_{\tau }\right)\right)$. Substituting (1) in (47), the above expression can be calculated as:

(48) $$\begin{array}{ll} \mu_{\gamma_{SEC}}(q) & =\frac{P_{1}B(m+q,n-q)}{B(m,n)\Lambda^{q}}+\frac{P_{3}\gamma_{\tau }^{q-n}}{B(m,n)\Lambda^{n}(n-q)}\\ & \times {}_{2}F_{1}(m+n,n-q;n-q+1;-1/\gamma_{\tau }\Lambda ). \end{array}$$

Likewise, the corresponding MGF of $\gamma_{SEC}$ can be expressed as:

(49) $$\begin{array}{ll} MGF_{\gamma_{SEC}}(s) & =P_{1}\int_{0}^{\infty }\exp(-s\gamma )f_{\gamma }(\gamma )d\gamma +P_{3}\int_{\gamma_{\tau }}^{\infty }\exp(-s\gamma )f_{\gamma }(\gamma )d\gamma \\ & =\frac{\left(P_{1}+P_{3})G_{1,2}^{2,1}[s/\Lambda |{}_{0,n}^{1-m}\right]}{\Gamma (m)\Gamma (n)}-\frac{P_{3}\Lambda^{m}\gamma_{\tau }^{m}}{\Gamma (m)\Gamma (n)}G_{1,1:0,1:1,1}^{0,1:1,0:1,1}[s\gamma_{\tau },\gamma_{\tau }\Lambda |{}_{-m}^{1-m}|{}_{0}^{-}|{}_{0}^{1-(m+n)}]. \end{array}$$

### 4.2. SECps Receiver

The PDF of the instantaneous SNR, $\;\gamma_{SECps}$, of the SECps can be written as [4]:

(50) $$f_{\gamma_{SECps}}(\gamma )=\{\begin{array}{ll} L{\left[F_{\gamma }(\gamma )\right]}^{L-1}f_{\gamma }(\gamma ), & \gamma \leq \gamma_{\tau };\\ P_{2}f_{\gamma }(\gamma ), & \gamma >\gamma_{\tau }, \end{array}$$

where $f_{\gamma }\left(\gamma \right)$ and $F_{\gamma }\left(\gamma \right)$ can be obtained by using (1) and (2), respectively. By integrating the expression of $f_{\gamma_{SECps}}\left(\gamma \right)$ in (50) with respect to  γ, the corresponding CDF of $\gamma_{SECps}$ can be written as:

(51) $$F_{\gamma_{SECps}}(\gamma )=\{\begin{array}{cc} {\left[F_{\gamma }(\gamma )\right]}^{L}, & \gamma \leq \gamma_{\tau };\\ P_{2}F_{\gamma }(\gamma ), & \gamma >\gamma_{\tau }. \end{array}$$

Similar to (41), the *q*th moments of $\gamma_{SECps}$ can be given as:

(52) $$\mu_{\gamma_{SECps}}(q)=\underset{I_{3}}{\underbrace{\int_{0}^{\gamma_{\tau }}\gamma^{q}L{\left[F_{\gamma }(\gamma )\right]}^{L-1}f_{\gamma }(\gamma )d\gamma }}+\underset{I_{4}}{\underbrace{P_{2}\int_{\gamma_{\tau }}^{\infty }\gamma^{q}f_{\gamma }(\gamma )d\gamma }}.$$

In order to calculate the integral *I*_3_ in (52), we first express (1) and (2) by using the univariate Fox’s H-function with the aid of ([47], Equation (1.126)) and ([47], Equation (1.132)) as follows:

(53) $$f_{\gamma }(\gamma )=\frac{\Lambda^{m}\gamma^{m-1}}{\Gamma (m)\Gamma (n)}H_{1,1}^{1,1}[\Lambda \gamma |{}_{\left(0,1\right)}^{\left(1-m-n,1\right)}],$$

(54) $$F_{\gamma }(\gamma )=\frac{{\left(\Lambda \gamma \right)}^{m}}{\Gamma (m)\Gamma (n)}H_{2,2}^{1,2}[\Lambda \gamma |{}_{\left(0,1),(-m,1\right)}^{\left(1-m-n,1),(1-m,1\right)}],$$

Then, by utilizing the Mellin–Barnes-type integral of the univariate H-function defined in ([47], Equation (1.2)) and substituting (53) and (54) in the integral term *I*_3_, *I*_3_ can be expressed as:

(55) $$I_{3}=\frac{\Lambda^{Lm}}{\Gamma^{L}(m)\Gamma^{L}(n)}{\left(\frac{1}{2\pi j}\right)}^{L}\int_{L_{1}}\cdots \int_{L_{L}}\left(\Delta_{1}\Delta_{2}\right)\Lambda^{-\sum_{l=1}^{L}t_{l}}dt_{1}\cdots dt_{L},$$

where $\Delta_{1}=\int_{0}^{\gamma_{\tau }}\gamma^{Lm-\sum_{l=1}^{L}t_{l}+q-1}d\gamma ,\Delta_{2}=L(m-t_{L})\prod_{l=1}^{L}\frac{\Gamma (t_{l})\Gamma (m+n-t_{l})\Gamma (m-t_{l})}{\Gamma (1+m-t_{l})}$, $j=\sqrt{-1}$, and $L_{l}$ denotes the *l*th appropriate contour, which starts at the point $\tau_{l}-j\infty$ and goes to the point $\tau_{l}-j\infty$ with $\tau_{l}$ as a constant value, l∈{1,2,…,L}.

To this effect, substituting the solution of $\Delta_{1}$ into (55) and employing the multiple Mellin–Barnes-type contour integral of the multivariate Fox’s H-function defined in ([47], Equation (A.1)), the compact form of *I*_3_ can be re-expressed as:

(56) $$I_{3}=\frac{L\Lambda^{Lm}\gamma_{{}_{\tau }}^{Lm+q}}{\Gamma^{L}(m)\Gamma^{L}(n)}H_{1,1:{\left[2,2\right]}_{l=1:L-1}:1,1}^{0,1:{\left[1,2\right]}_{l=1:L-1}:1,1}[\overset{L}{\overbrace{\gamma_{\tau }\Lambda ,\cdots ,\gamma_{\tau }\Lambda }}|{}_{\left(-Lm-q;{\left\{1\right\}}_{l=1:L}\right)}^{\left(1-Lm-q;{\left\{1\right\}}_{l=1:L}\right)}|{}_{{\left[(0,1),(-m,1)\right]}_{l=1:L-1}}^{{\left[(1-m-n,1),(1-m,1)\right]}_{l=1:L-1}}|{}_{\left(0,1\right)}^{\left(1-m-n,1\right)}].$$

where $H_{p,q:p_{1},q_{1}:\cdots :p_{L},q_{L}}^{m,n:m_{1},n_{1}:\cdots :m_{L},n_{L}}\left[\cdot \right]$ denotes a multivariate Fox’s H-function defined in ([47], Equation (A.1)). Similar to (48), the integral term *I*_4_ can be obtained as:

(57) $$I_{4}=\frac{P_{2}\gamma_{\tau }^{q-n}\Lambda^{-n}}{B(m,n)(n-q)}{}_{2}F_{1}(m+n,n-q;n-q+1;-1/\gamma_{\tau }\Lambda ).$$

Finally, by inserting (56) and (57) into (52), the closed-form expression of the *q*th moments of $\gamma_{SECps}$ can be obtained.

By performing a similar procedure yielding (56) and (57), and with the help of ([43], Equation (3.381.1)) and ([49], Equation (06.06.07.002.01)), after mathematical manipulation, the corresponding MGF of $\gamma_{SECps}$ can be written as:

(58) $$\begin{array}{ll} MGF_{\gamma_{SECps}}(s) & =\frac{L{\left(\Lambda /s\right)}^{Lm}}{\Gamma^{L}(m)\Gamma^{L}(n)}H_{1,0:{\left[2,2\right]}_{l=1:L-1}:1,1:1,1}^{0,1:{\left[1,2\right]}_{l=1:L-1}:1,1:0,1}[\overset{L}{\overbrace{\frac{\Lambda }{s},\cdots ,\frac{\Lambda }{s}}},s\gamma_{\tau }|{}_{-}^{\left(1-Lm;{\left\{1\right\}}_{l=1:L},-1\right)}|{}_{{\left[(0,1),(-m,1)\right]}_{l=1:L-1}}^{{\left[(1-m-n,1),(1-m,1)\right]}_{l=1:L-1}}|{}_{\left(0,1\right)}^{\left(1-m-n,1\right)}|{}_{\left(0,1\right)}^{\left(1,1\right)}]\\ & +\frac{P_{2}G_{1,2}^{2,1}[s/\Lambda |{}_{0,n}^{1-m}]}{\Gamma (m)\Gamma (n)}-\frac{P_{2}\Lambda^{m}\gamma_{\tau }^{m}}{\Gamma (m)\Gamma (n)}G_{1,1:0,1:1,1}^{0,1:1,0:1,1}[s\gamma_{\tau },\gamma_{\tau }\Lambda |{}_{-m}^{1-m}|{}_{0}^{-}|{}_{0}^{1-(m+n)}]. \end{array}$$

## 5. Performance Analysis and Optimization

In this section, by applying the previous statistical characteristics of the output SNR for SSC, SEC, and SECps schemes, various performance measures of wireless communication, such as the OP, the average SNR, the AoF, the ABEP/ASEP, and the average capacity, are evaluated.

### 5.1. Outage Probability

The OP is an important performance metric of wireless communications systems operating over fading channels. It is defined as the probability that the instantaneous SNR at the receiver output, *γ* falls below a predefined outage threshold, *γ_th_*. Based on this definition, the outage probability of SSC over Fisher–Snedecor F fading channels can be mathematically expressed as [1]:

(59) $$P_{out}=\mathrm{Pr}(0<\gamma_{SSC}<\gamma_{th})=\int_{0}^{\gamma_{th}}f_{\gamma_{SSC}}(\gamma )d\gamma .$$

Therefore, the corresponding OP is readily deduced as follows:

(60) $$P_{out}=F_{\gamma_{SSC}}(\gamma_{th}),$$

where $F_{\gamma_{SCC}}\left(\gamma_{th}\right)$ can be obtained by using (25)–(28) and replacing *γ* with *γ_th_* over different fading conditions. Similarly, we can also obtain the OP of SEC and SECps by using (46) and (51) over i.i.d. fading channels.

In order to find the optimal switching threshold ($\gamma_{\tau }^{*}$) with the minimum outage probability, the outage threshold (*γ_th_*) is usually chosen as $\gamma_{\tau }^{*}$ [1], namely $\gamma_{\tau }^{*}$ = *γ_th_*. Thus, the optimal outage performance of SCC receivers in any fading channels is reduced to the performance of the PSC receivers.

### 5.2. Average Output SNR and Amount of Fading

#### 5.2.1. Average Output SNR

The average output SNR is a useful performance measure serving as an excellent indicator of the overall system’s fidelity. The average output SNR of SSC over various Fisher–Snedecor F fading channels can be obtained by setting *q* = 1 in (38) (or (43) and (44)); that is, ${\bar{\gamma }}_{SCC}=\mu_{\gamma_{SCC}}\left(1\right)$.

To get the optimum switching threshold that maximizes the average output SNR, by differentiating $\mu_{\gamma_{SCC}}\left(1\right)$ in (38) with respect to $\gamma_{\tau }$ and with some mathematical manipulation, we have:

(61) $$\frac{\partial }{\partial \gamma_{\tau }}\mu_{\gamma_{ssc}}(1)=B(\gamma_{\tau })({ϒ}_{1}-{ϒ}_{2})+\left(P/F_{1}\right)(\Phi_{1}(\gamma_{\tau })-\Phi_{2}(\gamma_{\tau }))+\Phi_{2}(\gamma_{\tau }),$$

where:

(62a) $$B(\cdot )=\frac{f_{\gamma_{2}}(\cdot )F_{1}-f_{\gamma_{1}}(\cdot )F_{2}}{{\left(F_{1}+F_{2}\right)}^{2}}$$

(62b) $$\Phi_{i}(\cdot )=\int_{0}^{\infty }\gamma f_{\gamma_{1},\gamma_{2}}(\cdot ,\gamma )d\gamma -\gamma_{\tau }f_{\gamma_{i}}(\cdot )$$

(62c) $${ϒ}_{i}=\int_{0}^{\infty }\gamma g_{\gamma_{i}}(\gamma )d\gamma +\int_{\gamma_{\tau }}^{\infty }\gamma f_{\gamma_{i}}(\gamma )d\gamma$$

We substitute (1), (13), (19) and (20) into (62), and then set the final results of (61) to zero. For this general case, it is hard to find the analytical solution of the optimum value $\gamma_{\tau }^{*}$. However, this solution can be evaluated by using the numerical method with the help of the MATLAB software package or Mathematica tool. For the i.i.d. case, the closed-form expression of the optimum switching threshold for $\mu_{\gamma_{SCC}}\left(q\right)$ can be obtained as:

(63) $$\gamma_{\tau }^{*}={\left(\frac{B(m+q,n-q)}{B(m,n)\Lambda^{q}}\right)}^{1/q},$$

where $\mu_{\gamma_{SCC}}\left(q\right)\;$ has the maximum value. When *q* = 1, $\gamma_{\tau }^{*}=\bar{\gamma }$, the maximum average output SNR at the SSC can be written as:

(64) $${\bar{\gamma }}_{ssc}=\bar{\gamma }\left(\frac{m^{m-1}}{B(m,n){\left(n-1\right)}^{m}}{}_{2}F_{1}(m+n,m;m+1;-\frac{m}{n-1})+\frac{{\left(n-1\right)}^{n-1}}{B(m,n)m^{n}}{}_{2}F_{1}(m+n,n-1;n;-\frac{n-1}{m})\right).$$

Based on (48), the average output SNR at the SEC can also readily obtained by setting *q* = 1. To obtain the optimum switching threshold $\gamma_{\tau }^{*}$, the derivative of (47) with respect to $\gamma_{\tau }$ when *q* = 1 can be expressed as:

(65) $$\begin{array}{ll} \frac{\partial \mu_{\gamma_{SEC}}(1)}{\partial \gamma_{\tau }} & =(L-1)F{\left(\gamma_{\tau }\right)}^{L-2}f_{\gamma }(\gamma_{\tau })\int_{0}^{\infty }\gamma f_{\gamma }(\gamma )d\gamma \\ & +\sum_{i=0}^{L-2}\left(iF{\left(\gamma_{\tau }\right)}^{i-1}f_{\gamma }(\gamma_{\tau })\int_{\gamma_{\tau }}^{\infty }\gamma f_{\gamma }(\gamma )d\gamma +\gamma_{\tau }F{\left(\gamma_{\tau }\right)}^{i}f_{\gamma }(\gamma_{\tau })\right). \end{array}$$

In order to simplify the analysis process, we apply the method in [50] and let $\gamma_{\tau }^{*}=\alpha \bar{\gamma }$, where α is a function of *L* and $\bar{\gamma }$. By inserting $\gamma_{\tau }^{*}=\alpha \bar{\gamma }$ into (65), the average output SNR at the SEC can be given over Fisher–Snedecor F fading channels as:

(66) $${\bar{\gamma }}_{SEC}=\bar{\gamma }\left(P_{1}+\frac{P_{3}{\left(\left(n-1\right)/\alpha \right)}^{n-1}}{B(m,n)m^{n}}{}_{2}F_{1}(m+n,n-1;n;-\left(n-1\right)/\alpha m)\right).$$

In Table 3, it can be observed that the value of α increases as *L* grows, regardless of the fading conditions. However, the value of α gets smaller with the increase of the values of the shadowing parameters (*n*), and it approaches that of the case of Nakagami-m (*n* → ∞) in [50].

#### 5.2.2. Amount of Fading

The AoF is the critical performance measure indicating the severity of fading for the wireless communication system, which is typically independent of the average fading power and can be utilized to parameterize the distribution of the SNR of the received signal. Based on the definition, the AoF of SSC over Fisher–Snedecor F fading channels is given as follows:

(67) $$AoF=\mu_{\gamma_{SSC}}(2)/\mu_{\gamma_{SSC}}^{2}(1)-1.$$

By setting *q* = 1 and 2 in (40) and (41), respectively, then inserting them in (38), the analytical expression of AoF can be deduced over Fisher–Snedecor composite fading channels. Similarly, the analytical expression of AoF for SEC and SECps can also be easily obtained. The AoF can also be used to study the spectral efficiency and the channel quality estimation index.

### 5.3. Average BEP/SEP

The ABEP/ASEP is an alternative important measure for performance analysis of wireless digital communications systems operating in fading environments. In this section, by using the previously derived MGF formulas for SSC, SEC, and SECps, we consider the ABEP/ASEP including both coherent and non-coherent modulation schemes over Fisher–Snedecor F fading channels.

#### 5.3.1. ABEP for NCBFSK and BDPSK

For non-coherent binary frequency shift keying (NCBFSK) and binary differential phase-shift keying (BDPSK), *P_e_*(E) can be expressed as [1]:

(68) $$P_{e}(E)=aMGF_{\gamma_{SSC}}(b),$$

where a and *b* are the constants, depending on the specific modulation schemes. In particular, a = 0.5, *b* = 0.5 for NCBFSK; and a = 0.5, *b* = 1 for BDPSK.

By substituting (29), (33), (35) and (37) into (68), we can obtain the ABEP of NCFSK and BDPSK for SSC in various fading conditions. Similarly, the ABEP of NCFSK and BDPSK for SEC and SECps can be obtained by using (49) and (58), respectively.

In order to find the optimum switching threshold and avoid the complicated calculations, we only consider the identical distribution case of the SSC. By using (32), we differentiate (68) with respect to $\gamma_{\tau }$ and set the result to zero, whereby the optimum switching threshold can be obtained by solving the following formula:

(69) $$\int_{0}^{\infty }\exp(-b\gamma )f_{\gamma_{1},\gamma_{2}}(\gamma ,\gamma_{\tau }^{*})d\gamma =\exp(-b\gamma_{\tau }^{*})f_{\gamma_{2}}(\gamma_{\tau }^{*}).$$

As the two branches are independent of each other, the above formula reduces to:

(70) $$\int_{0}^{\infty }\exp(-b\gamma )f(\gamma )d\gamma =\exp(-b\gamma_{\tau }^{*}).$$

Then, the solution of the optimum switching threshold can be given over Fisher–Snedecor F fading channels as:

(71) $$\gamma_{\tau }^{*}=-\frac{1}{b}\ln\left(\frac{G_{1,2}^{2,1}[s/\Lambda |{}_{0,n}^{1-m}]}{\Gamma (m)\Gamma (n)}\right).$$

For the multiple-branch SEC receiver, by substituting (49) into (68) and differentiating (68) with respect to $\gamma_{\tau }$, then letting $\partial P_{e}\left(E\right)/\partial \gamma_{\tau }{|}_{\gamma_{\tau }^{*}=\gamma_{\tau }}=0$ with some mathematical manipulation, one equation as a function of $\gamma_{\tau }$ can be expressed as:

(72) $$(L-1)F{\left(\gamma_{\tau }\right)}^{L-2}{ϒ}_{3}+\sum_{i=0}^{L-2}\left(iF{\left(\gamma_{\tau }\right)}^{i-1}{ϒ}_{4}+\gamma_{\tau }F{\left(\gamma_{\tau }\right)}^{i}\right)=0.$$

where ${ϒ}_{3}=\frac{G_{1,2}^{2,1}[s/\Lambda |{}_{0,n}^{1-m}]}{\Gamma (m)\Gamma (n)}$, ${ϒ}_{4}=\frac{G_{1,2}^{2,1}[s/\Lambda |{}_{0,n}^{1-m}]}{\Gamma (m)\Gamma (n)}-\frac{\Lambda^{m}\gamma_{\tau }^{m}}{\Gamma (m)\Gamma (n)}G_{1,1:0,1:1,1}^{0,1:1,0:1,1}[s\gamma_{\tau },\gamma_{\tau }\Lambda |{}_{-m}^{1-m}|{}_{0}^{-}|{}_{0}^{1-(m+n)}]$.

To find the optimal solution in (72), one numerical search method is required using the MATLAB software package.

#### 5.3.2. ABEP for BPSK and BFSK 

For coherent modulation schemes, such as BPSK, BFSK, and BFSK with minimum correlation, the ABEP at the output of a dual-branch SSC system can be expressed by applying the MGF method as [1]:

(73) Pe(E)=1π∫0π2MGFγSSC(gsin2θ)dθ.

where *g* = 1 for BPSK, *g* = 0.5 for BFSK, and *g* = 0.715 for BFSK with minimum correlation.

Here, only identically distributed fading is considered for feasible computation. By substituting (33) in (73) and applying the definition of the bivariate Meijer G-function, and after some mathematical manipulation, we can obtain the ABEP values of BPSK, BFSK, and BFSK with minimum correlation for SSC over correlated and identical Fisher–Snedecor composite fading channels as (the mathematical derivation is provided in Appendix B):

(74) $$\begin{array}{ll} P_{e}(E) & =\sum_{k=0}^{\infty }\sum_{l=0}^{\infty }\frac{\rho_{N}^{k}\rho_{G}^{l}\phi {\left(\gamma_{\tau }\eta_{2}\right)}^{m+k}{}_{2}F_{1}[\lambda ,m+k;1+m+k,-\eta_{2}\gamma_{\tau }]}{l!k!2\sqrt{\pi }\Gamma (m)\Gamma (n)B(k+m,l+n)(m+k)}G_{2,3}^{3,1}[g/\eta_{1}|{}_{0,n+l,0.5}^{1-(m+k),1}]\\ & +\frac{G_{2,3}^{3,1}[g/\Lambda_{2}|{}_{0,n,0.5}^{1-m,1}]}{2\sqrt{\pi }\Gamma (m)\Gamma (n)}-\frac{\Lambda_{2}^{m}\gamma_{\tau }^{m}}{2\sqrt{\pi }\Gamma (m)\Gamma (n)}G_{1,1:1,2:1,1}^{0,1:2,0:1,1}[g\gamma_{\tau },\gamma_{\tau }\Lambda_{2}|{}_{-m}^{1-m}|{}_{0,0.5}^{1}|{}_{0}^{1-(m+n)}]. \end{array}$$

For the non-correlated case, Equation (74) can be simplified as:

(75) $$P_{e}(E)=\frac{\left(1+F_{\gamma }(\gamma_{\tau }))G_{2,3}^{3,1}[g/\Lambda |{}_{0,n,0.5}^{1-m,1}\right]}{2\sqrt{\pi }\Gamma (m)\Gamma (n)}-\frac{\Lambda^{m}\gamma_{\tau }^{m}}{2\sqrt{\pi }\Gamma (m)\Gamma (n)}G_{1,1:1,2:1,1}^{0,1:2,0:1,1}[g\gamma_{\tau },\gamma_{\tau }\Lambda |{}_{-m}^{1-m}|{}_{0,0.5}^{1}|{}_{0}^{1-(m+n)}].$$

To obtain the optimum switching threshold minimizing the ABEP of the above coherent modulation schemes, by inserting (36) in (73) and differentiating with respect to $\gamma_{\tau }$, then setting $\partial P_{e}\left(E\right)/\partial \gamma_{\tau }{|}_{\gamma_{\tau }^{*}=\gamma_{\tau }}=0$, we have:

(76) $$\gamma_{\tau }^{*}=\frac{1}{2g}{\left[Q^{-1}\left(\frac{G_{2,3}^{3,1}[g/\Lambda |{}_{0,n,0.5}^{1-m,1}]}{2\sqrt{\pi }\Gamma (m)\Gamma (n)}\right)\right]}^{2},$$

where *Q*^−1^(x) is the inverse function of the Gaussian *Q*-function defined in ([1], Equation (4.2)).

Similar to (63), the ABEP of coherent modulation schemes for SEC can also be obtained by substituting (49) into (73) and following the approach in Appendix B, as follows:

(77) $$P_{e}(E)=\frac{P_{1}+P_{3}}{2\sqrt{\pi }\Gamma (m)\Gamma (n)}G_{2,3}^{3,1}[g/\Lambda |{}_{0,n+l,0.5}^{1-(m+k),1}]-\frac{P_{3}\Lambda^{m}\gamma_{\tau }^{m}}{2\sqrt{\pi }\Gamma (m)\Gamma (n)}G_{1,1:1,2:1,1}^{0,1:2,0:1,1}[g\gamma_{\tau },\gamma_{\tau }\Lambda |{}_{-m}^{1-m}|{}_{0,0.5}^{1}|{}_{0}^{1-(m+n)}].$$

In order to find the ABEP values of coherent modulation schemes for SECps, we employ the multiple Mellin–Barnes-type contour integral of the multivariate Fox’s H-function and the approach in Appendix B again. By plugging (58) in (73), after some algebraic manipulation, the exact and novel expression of the ABEP for SECps can be derived as:

(78) $$\begin{array}{ll} P_{e}(E) & =\frac{L{\left(\Lambda /g\right)}^{Lm}}{2\sqrt{\pi }\Gamma^{L}(m)\Gamma^{L}(n)}H_{2,1:{\left[2,2\right]}_{l=1:L-1}:1,1:1,1}^{0,2:{\left[1,2\right]}_{l=1:L-1}:1,1:0,1}[\overset{L}{\overbrace{\frac{\Lambda }{g},\cdots ,\frac{\Lambda }{g}}},g\gamma_{\tau }|{}_{\left(-Lm;{\left\{1\right\}}_{l=1:L},-1\right)}^{\left(0.5-Lm;{\left\{1\right\}}_{l=1:L},-1),(1-Lm;{\left\{1\right\}}_{l=1:L},-1\right)}|{}_{{\left[(0,1),(-m,1)\right]}_{l=1:L-1}}^{{\left[(1-m-n,1),(1-m,1)\right]}_{l=1:L-1}}|{}_{\left(0,1\right)}^{\left(1-m-n,1\right)}|{}_{\left(0,1\right)}^{\left(1,1\right)}]\\ & +\frac{P_{2}}{2\sqrt{\pi }\Gamma (m)\Gamma (n)}G_{2,3}^{3,1}[g/\Lambda |{}_{0,n,0.5}^{1-m,1}]-\frac{P_{2}\Lambda^{m}\gamma_{\tau }^{m}}{2\sqrt{\pi }\Gamma (m)\Gamma (n)}G_{1,1:1,2:1,1}^{0,1:2,0:1,1}[g\gamma_{\tau },\gamma_{\tau }\Lambda |{}_{-m}^{1-m}|{}_{0,0.5}^{1}|{}_{0}^{1-(m+n)}]. \end{array}$$

#### 5.3.3. ASEP for MQAM

In this section, only the ASEP of MQAM for SSC is considered as an example for easier derivation. Hence, by using the MGF-based method, the ASEP of MQAM for SSC can be given as [1]:

(79) Pe(E)=4cπ∫0π2MGFγSSC(gQAMsin2θ)dθ⏟I5−4c2π∫0π4MGFγSSC(gQAMsin2θ)dθ⏟I6,

where $c=1-1/\sqrt{M},$ and $g_{\mathrm{QAM}}=3/2\left(M-1\right)$, *M* = 4, 16, …. For the first integral term $I_{5}$ in (79), we utilize the same methods provided in Appendix B. After some mathematical manipulation, we obtain:

(80) $$\begin{array}{ll} I_{5} & =\sum_{k=0}^{\infty }\sum_{l=0}^{\infty }\frac{2c\rho_{N}^{k}\rho_{G}^{l}\phi {\left(\gamma_{\tau }\eta_{2}\right)}^{m+k}{}_{2}F_{1}[\lambda ,m+k;1+m+k,-\eta_{2}\gamma_{\tau }]}{l!k!\sqrt{\pi }\Gamma (m)\Gamma (n)B(k+m,l+n)(m+k)}G_{2,3}^{3,1}[g_{QAM}/\eta_{1}|{}_{0,n+l,0.5}^{1-(m+k),1}]\\ & \frac{2cG_{2,3}^{3,1}[g_{QAM}/\Lambda_{2}|{}_{0,n,0.5}^{1-m,1}]}{\sqrt{\pi }\Gamma (m)\Gamma (n)}-\frac{2c\Lambda_{2}^{m}\gamma_{\tau }^{m}}{\sqrt{\pi }\Gamma (m\Gamma (n)}G_{1,1:1,2:1,1}^{0,1:2,0:1,1}[g_{QAM}\gamma_{\tau },\gamma_{\tau }\Lambda_{2}|{}_{-m}^{1-m}|{}_{0,0.5}^{1}|{}_{0}^{1-(m+n)}]. \end{array}$$

For the second integral term $I_{6}$ in (79), similar steps are considered as in Appendix B. We use the definition of the multivariable Fox’s H-function again in terms of the multiple Mellin–Barnes-type contour integral in ([47], Equation (A.1)), and provide the detailed derivative in Appendix C. Hence, the solution of $I_{6}$ can be given as:

(81) $$\begin{array}{ll} I_{6} & =\sum_{k=0}^{\infty }\sum_{l=0}^{\infty }\frac{\sqrt{2}c^{2}\rho_{N}^{k}\rho_{G}^{l}\phi {\left(\gamma_{\tau }\eta_{2}\right)}^{m+k}{}_{2}F_{1}[\lambda ,m+k;1+m+k,-\eta_{2}\gamma_{\tau }]}{l!k!\pi \sqrt{\pi }\Gamma (m)\Gamma (n)B(k+m,l+n)(m+k)}H_{1,1:1,2:1,1}^{0,1:2,1:1,1}[\frac{2g_{QAM}}{\eta_{1}},-0.5|{}_{\left(-0.5:-1,1\right)}^{\left(0.5:-1,1\right)}|{}_{\left(0:1),(n+l:1\right)}^{\left(1-(m+k):1\right)}|{}_{\left(0:1\right)}^{\left(0.5:1\right)}]\\ & +\frac{\sqrt{2}c^{2}}{\pi \sqrt{\pi }\Gamma (m)\Gamma (n)}H_{1,1:1,2:1,1}^{0,1:2,1:1,1}[\frac{2g_{QAM}}{\Lambda_{2}},-0.5|{}_{\left(-0.5:-1,1\right)}^{\left(0.5:-1,1\right)}|{}_{\left(0:1),(n:1\right)}^{\left(1-m:1\right)}|{}_{\left(0:1\right)}^{\left(0.5:1\right)}]\\ & -\frac{\sqrt{2}c^{2}\Lambda_{2}^{m}\gamma_{\tau }^{m}}{\pi \sqrt{\pi }\Gamma (m\Gamma (n)}H_{2,2:0,1:1,1:1,1}^{0,2:1,0:1,1:1,1}[2g_{QAM}\gamma_{\tau },\gamma_{\tau }\Lambda_{2},-0.5|{}_{\left(-0.5:-1,0,1),(-m:1,1,0\right)}^{\left(0.5:-1,0,1),(1-m:1,1,0\right)}|{}_{\left(0:1\right)}^{-}|{}_{\left(0:1\right)}^{\left(1-m-n:1\right)}|{}_{\left(0:1\right)}^{\left(0.5:1\right)}], \end{array}$$

where $H_{p,q:p_{1},q_{1}:p_{2},q_{2}}^{m,n:m_{1},n_{1}:m_{2},n_{2}}\left[\cdot \right]$ denotes a bivariate Fox’s H-function and $H_{p,q:p_{1},q_{1}:p_{2},q_{2}:p_{3},q_{3}}^{m,n:m_{1},n_{1}:m_{2},n_{2}:m_{3},n_{3}}\left[\cdot \right]$ denotes a triple-variable Fox’s H-function.

By inserting (80) and (81) into (79), the exact analytical expression of the ASEP of MQAM for SSC over correlated Fisher–Snedecor F composite fading can be obtained. For the independent case, by substituting (37) into (79) and with some manipulation, the exact closed-form expression of the ASEP of MQAM for SSC can be written as:

(82) $$\begin{array}{ll} P_{e}(E) & =\frac{2c(1+F_{\gamma }(\gamma_{\tau }))}{\sqrt{\pi }\Gamma (m)\Gamma (n)}\left(G_{2,3}^{3,1}[g_{QAM}/\Lambda_{2}|{}_{0,n,0.5}^{1-m,1}]-\frac{c}{\sqrt{2}\pi }H_{1,1:1,2:1,1}^{0,1:2,1:1,1}[\frac{2g_{QAM}}{\Lambda_{2}},-0.5|{}_{(-0.5:-1,1}^{(0.5:-1,1}|{}_{\left(0:1),(n:1\right)}^{\left(1-m:1\right)}|{}_{\left(0:1\right)}^{\left(0.5:1\right)}]\right)\\ & -\frac{2c\Lambda_{2}^{m}\gamma_{\tau }^{m}}{\sqrt{\pi }\Gamma (m\Gamma (n)}\left(\begin{array}{l} G_{1,1:1,2:1,1}^{0,1:2,0:1,1}[g_{QAM}\gamma_{\tau },\gamma_{\tau }\Lambda_{2}|{}_{-m}^{1-m}|{}_{0,0.5}^{1}|{}_{0}^{1-(m+n)}]\\ -\frac{c}{\sqrt{2}\pi }H_{2,2:0,1:1,1:1,1}^{0,2:1,0:1,1:1,1}[2g_{QAM}\gamma_{\tau },\gamma_{\tau }\Lambda_{2},-0.5|{}_{\left(-0.5:-1,0,1),(-m:1,1,0\right)}^{\left(0.5:-1,0,1),(1-m:1,1,0\right)}|{}_{\left(0,1\right)}^{-}|{}_{\left(0:1\right)}^{\left(1-m-n:1\right)}|{}_{\left(0:1\right)}^{\left(0.5:1\right)}] \end{array}\right). \end{array}$$

By inserting (36) into (79) and adopting a similar approach as in (76), then finding a root of a quadratic equation $cQ^{2}\left(\sqrt{2g_{QAM}\gamma_{\tau }}\right)-Q\left(\sqrt{2g_{QAM}\gamma_{\tau }}\right)+\Delta =0$, we can get a closed-form expression of the optimum switching threshold of MQAM for SSC over Fisher–Snedecor F composite fading as:

(83) $$\gamma_{\tau }^{*}=\frac{1}{2g_{QAM}}{\left[Q^{-1}\left(\frac{1-\sqrt{1-4c\Delta }}{2c}\right)\right]}^{2},$$

where: $\Delta =\frac{(2\sqrt{\pi }{)}^{-1}}{\Gamma (m)\Gamma (n)}\left(G_{2,3}^{3,1}[\frac{g_{QAM}}{\Lambda }|{}_{0,n,0.5}^{1-m,1}]-\frac{c}{\sqrt{2}\pi }H_{1,1:1,2:1,1}^{0,1:2,1:1,1}[\frac{2g_{QAM}}{\Lambda },-0.5|{}_{(-0.5:-1,1}^{(0.5:-1,1}|{}_{\left(0:1),(n:1\right)}^{\left(1-m:1\right)}|{}_{\left(0:1\right)}^{\left(0.5:1\right)}]\right)$.

### 5.4. Ergodic Capacity

The channel capacity, in Shannon’s sense, is a core performance measure, since it provides the maximum achievable transmission rate in which the errors are recoverable. The ergodic capacity of the SSC in wireless fading channels can be expressed as [1]:

(84) $${\bar{C}}_{\gamma_{ssc}}=\frac{B}{\ln2}\int_{0}^{\infty }\ln(1+\gamma )f_{\gamma_{ssc}}(\gamma )d\gamma ,$$

where *B* denotes the bandwidth of the channel. By inserting (17) into (84) along with some manipulations the ergodic capacity of the SSC in the n.i.n.i.d. case can be written as:

(85) $${\bar{C}}_{\gamma_{ssc}}=\sum_{i=1}^{2}(P/F_{i}){\bar{C}}_{\gamma_{ssc}}^{\left(\gamma_{i}\right)}.$$

where:

(86) $${\bar{C}}_{\gamma_{ssc}}^{\left(\gamma_{i}\right)}=\int_{0}^{\infty }\ln(1+\gamma )g_{\gamma_{i}}(\gamma )d\gamma +\int_{\gamma_{\tau }}^{\infty }\ln(1+\gamma )f_{\gamma_{i}}(\gamma )d\gamma .$$

By substituting $g_{\gamma_{1}}\left(\gamma \right)$ in (19), $g_{\gamma_{2}}\left(\gamma \right)\;$ in (20), and $f_{\gamma_{i}}\left(\gamma \right)\;$ in (1) into (86), then applying Meijer G-function to represent ln(1+x) ([51], Equation (11)) and adopting the same steps as (A3) in Appendix A, after some algebraic manipulation, ${\bar{C}}_{\gamma_{SSC}}^{\left(\gamma_{1}\right)}$ and ${\bar{C}}_{\gamma_{SSC}}^{\left(\gamma_{2}\right)}\;$ are yielded, respectively, as:

(87) $$\begin{array}{ll} {\bar{C}}_{\gamma_{ssc}}^{\left(\gamma_{1}\right)} & =\sum_{k=0}^{\infty }\sum_{l=0}^{\infty }\sum_{i=0}^{\infty }\sum_{j=0}^{\infty }\frac{B\rho_{N}^{k+i}\rho_{G}^{l+j}\Theta \Gamma (\lambda_{1}){\left(m_{2}-m_{1}\right)}_{i}{\left(n_{2}-n_{1}\right)}_{j}\alpha_{1}^{m_{1}+k}\gamma_{\tau }^{m_{1}+k}}{k!l!i!j!\ln2\Gamma (m_{1})\Gamma (n_{1})\Gamma (m_{2}+k+i)\Gamma (n_{2}+l+j)(m_{1}+k)}\\ & \times G_{3,3}^{3,2}[\alpha_{2}|{}_{m_{2}+k+i,0,0}^{1-(n+l+j),0,1}]{}_{2}F_{1}[\lambda_{1},m_{1}+k;1+m_{1}+k,-\alpha_{1}\gamma_{\tau }]\\ & +\frac{BG_{3,3}^{3,2}[\Lambda_{1}|{}_{m_{1},0,0}^{1-n_{1},0,1}]}{\ln2\Gamma (m_{1})\Gamma (n_{1})}-\frac{B\Lambda_{1}^{m_{1}}\gamma_{\tau }^{m_{1}}}{\ln2\Gamma (m_{1})\Gamma (n_{1})}G_{1,1:2,2:1,1}^{0,1:1,2:1,1}[\gamma_{\tau },\gamma_{\tau }\Lambda_{1}|{}_{-m_{1}}^{1-m_{1}}|{}_{1,0}^{1,1}|{}_{0}^{1-(m_{1}+n_{1})}], \end{array}$$

(88) $$\begin{array}{ll} {\bar{C}}_{\gamma_{ssc}}^{\left(\gamma_{2}\right)} & =\sum_{k=0}^{\infty }\sum_{l=0}^{\infty }\sum_{i=0}^{\infty }\sum_{j=0}^{\infty }\frac{B\rho_{N}^{k+i}\rho_{G}^{l+j}\Theta {\left(m_{2}-m_{1}\right)}_{i}{\left(n_{2}-n_{1}\right)}_{j}\alpha_{2}^{m_{2}+k+i}\gamma_{\tau }^{m_{2}+k+i}G_{3,3}^{3,2}[\alpha_{1}|{}_{m_{1}+k,0,0}^{1-n_{1}-l,0,1}]}{k!l!i!j!\ln2B(m_{2}+k+i,n_{2}+l+j)\Gamma (m_{1})\Gamma (n_{1})(m_{2}+k+i)}\\ & \times {}_{2}F_{1}[\lambda_{2}+i+j,m_{2}+k+i;m_{2}+k+i+1;-\alpha_{2}\gamma_{\tau }]\\ & +\frac{BG_{3,3}^{3,2}[\Lambda_{2}|{}_{m_{2},0,0}^{1-n_{2},0,1}]}{\ln2\Gamma (m_{2})\Gamma (n_{2})}-\frac{B\Lambda_{2}^{m_{2}}\gamma_{\tau }^{m_{2}}}{\ln2\Gamma (m_{2})\Gamma (n_{2})}G_{1,1:2,2:1,1}^{0,1:1,2:1,1}[\gamma_{\tau },\gamma_{\tau }\Lambda_{2}|{}_{-m_{2}}^{1-m_{2}}|{}_{1,0}^{1,1}|{}_{0}^{1-(m_{2}+n_{2})}]. \end{array}$$

Based on (21) and (84), the ergodic capacity of the SSC over correlated and identical Fisher–Snedecor F composite fading channels can be expressed as:

(89) $${\bar{C}}_{\gamma_{ssc}}=\frac{B}{\ln2}\int_{0}^{\infty }\ln(1+\gamma )g_{\gamma_{2}}(\gamma )d\gamma +\frac{B}{\ln2}\int_{\gamma_{\tau }}^{\infty }\ln(1+\gamma )f_{\gamma_{2}}(\gamma )d\gamma .$$

By substituting (22) and (1) into (89) and applying the same approach as in (88), the analytical expression of (89) can be obtained as:

(90) $$\begin{array}{ll} {\bar{C}}_{\gamma_{ssc}} & =\sum_{k=0}^{\infty }\sum_{l=0}^{\infty }\frac{B\rho_{N}^{k}\rho_{G}^{l}\phi {\left(\eta_{2}\gamma_{\tau }\right)}^{m+k}G_{3,3}^{3,2}[\eta_{1}|{}_{(m+k),0,0}^{1-(n+l),0,1}]}{\ln2l!k!\Gamma (m)\Gamma (n)B(k+m,l+n)(m+k)}\\ & \times {}_{2}F_{1}[\lambda ,m+k;1+m+k,-\eta_{2}\gamma_{\tau }]\\ & +\frac{BG_{3,3}^{3,2}[\Lambda_{2}|{}_{m,0,0}^{1-n,0,1}]}{\ln2\Gamma (m)\Gamma (n)}-\frac{B\Lambda_{2}^{m}\gamma_{\tau }^{m}}{\ln2\Gamma (m)\Gamma (n)}G_{1,1:2,2:1,1}^{0,1:1,2:1,1}[\gamma_{\tau },\gamma_{\tau }\Lambda_{2}|{}_{-m}^{1-m}|{}_{1,0}^{1,1}|{}_{0}^{1-(m+n)}]. \end{array}$$

While for the i.i.d. case, Equation (89) can be further simplified as:

(91) $${\bar{C}}_{\gamma_{ssc}}=\frac{B[F_{\gamma }(\gamma_{\tau })+1]}{\ln2}\int_{0}^{\infty }\ln(1+\gamma )f_{\gamma }(\gamma )d\gamma -\frac{B}{\ln2}\int_{0}^{\gamma_{\tau }}\ln(1+\gamma )f_{\gamma }(\gamma )d\gamma .$$

By applying the same steps as (A3) in Appendix A, the closed-form expression of the ergodic capacity of the SSC in the i.i.d case can be obtained as:

(92) $${\bar{C}}_{\gamma_{ssc}}=\frac{BG_{3,3}^{3,2}[\Lambda |{}_{m,0,0}^{1-n,0,1}][F_{\gamma }(\gamma_{\tau })+1]}{\ln2\Gamma (m)\Gamma (n)}-\frac{B\Lambda^{m}\gamma_{\tau }^{m}}{\ln2\Gamma (m)\Gamma (n)}G_{1,1:2,2:1,1}^{0,1:1,2:1,1}[\gamma_{\tau },\gamma_{\tau }\Lambda |{}_{-m}^{1-m}|{}_{1,0}^{1,1}|{}_{0}^{1-(m+n)}].$$

To obtain the optimum switching threshold so as to maximize the ergodic capacity $\;{\bar{C}}_{\gamma_{SCC}}$ in the i.i.d. case, we use (91) and set $\;\partial {\bar{C}}_{\gamma_{SCC}}/\partial \gamma_{\tau }{|}_{\gamma_{\tau }^{*}=\gamma_{\tau }}=0$. After some algebraic manipulation, the optimum switching threshold can be yielded as $\gamma_{\tau }^{*}=\exp[{ϒ}_{4}]-1$, where: $\gamma_{4}=\exp\left(\frac{G_{3,3}^{3,2}[\Lambda |{}_{m,0,0}^{1-n,0,1}]}{\Gamma (m)\Gamma (n)}\right)-1$.

For the multiple-branch SEC receiver, by substituting (45) into (84), the closed-form expression of ergodic capacity in the i.i.d. case can be expressed as:

(93) $$\begin{array}{ll} {\bar{C}}_{\gamma_{SEC}} & =\frac{BP_{1}}{\ln2}\int_{0}^{\infty }\ln(1+\gamma )f_{\gamma }(\gamma )d\gamma +\frac{BP_{3}}{\ln2}\int_{\gamma_{\tau }}^{\infty }\ln(1+\gamma )f_{\gamma }(\gamma )d\gamma \\ & =\frac{BG_{3,3}^{3,2}[\Lambda |{}_{m,0,0}^{1-n,0,1}]\left(P_{1}+P_{3}\right)}{\ln2\Gamma (m)\Gamma (n)}-\frac{BP_{3}\Lambda^{m}\gamma_{\tau }^{m}}{\ln2\Gamma (m)\Gamma (n)}G_{1,1:2,2:1,1}^{0,1:1,2:1,1}[\gamma_{\tau },\gamma_{\tau }\Lambda |{}_{-m}^{1-m}|{}_{1,0}^{1,1}|{}_{0}^{1-(m+n)}]. \end{array}$$

Similar to (78), by plugging (45) into (84), the closed-form expression of ergodic capacity for the multiple-branch SECps receiver in the i.i.d. case can be written as:

(94) $$\begin{array}{ll} {\bar{C}}_{\gamma_{SECps}} & =\frac{BL{\left(\Lambda \gamma_{\tau }\right)}^{Lm}}{\Gamma^{L}(m)\Gamma^{L}(n)\ln2}H_{1,1:{\left[2,2\right]}_{l=1:L-1}:1,1:2,2}^{0,1:{\left[1,2\right]}_{l=1:L-1}:1,1:1,2}[\overset{L}{\overbrace{\Lambda \gamma_{\tau },\cdots ,\Lambda \gamma_{\tau }}},\gamma_{\tau }|{}_{\left(-Lm;{\left\{1\right\}}_{l=1:L+1}\right)}^{\left(1-Lm;{\left\{1\right\}}_{l=1:L+1}\right)}|{}_{{\left[(0,1),(-m,1)\right]}_{l=1:L-1}}^{{\left[(1-m-n,1),(1-m,1)\right]}_{l=1:L-1}}|{}_{\left(0,1\right)}^{\left(1-m-n,1\right)}|{}_{\left(1,1),(0,1\right)}^{\left(1,1),(1,1\right)}]\\ & +\frac{P_{2}B}{\ln2\Gamma (m)\Gamma (n)}G_{3,3}^{3,2}[\Lambda |{}_{m,0,0}^{1-n,0,1}]-\frac{P_{2}B{\left(\Lambda \gamma_{\tau }\right)}^{m}}{\ln2\Gamma (m)\Gamma (n)}G_{1,1:2,2:1,1}^{0,1:1,2:1,1}[\gamma_{\tau },\gamma_{\tau }\Lambda |{}_{-m}^{1-m}|{}_{1,0}^{1,1}|{}_{0}^{1-(m+n)}]. \end{array}$$

## 6. Numerical Results and Discussions 

Capitalizing on the aforementioned derived analytical expressions, various numerical and simulation results under different Fisher–Snedecor F fading scenarios are presented and discussed in this section. In the simulations, we adopted the simulation approaches described in [29,38]. All of the simulation results show a good agreement with the numerical analysis and validate the accuracy of our derivations. In the theoretical analysis, a Python code provided in [52] was applied to evaluate the bivariate Meijer G-function and multivariate Fox’s H-function in Section 5. For certain analytical expressions, including the sum of the two-fold infinite series, the required minimum numbers of truncated terms are provided in Tables 1–4 in [38] for different cases in order to meet the given target accuracy, while for the sum of the four-fold infinite series in the n.i.n.i.d. case, an example for the convergence rate of the infinite sum by using (15) and (25) at the sixth significant figure is as follows: *k_min_* = *l_min_* = 30, *i_min_* = *j_min_* = 20, as *m*_1_ = 1, *m*_2_ = 2, *n*_1_ = 5, *n*_2_ = 6, $\rho_{N}=\rho_{G}=0.5$, $\gamma_{\tau }$ = $\gamma_{th}$ = 3 dB. Furthermore, we consider comparisons and discussions regarding the impacts of the correlation coefficients and the optimum threshold on the performance of the switched diversity systems, because the impacts of the multipath parameters and the shadowing parameters on the performance were widely discussed in [34,38].

**Figure 1:**
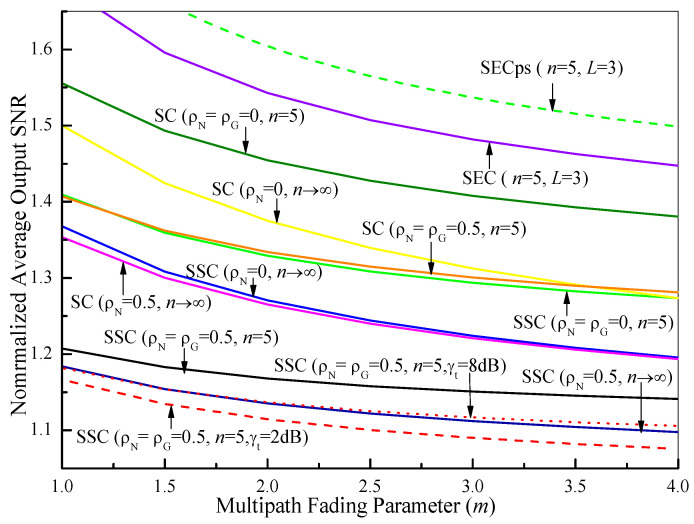
Comparison of the normalized average output SNR values of SCC (${\bar{\gamma }}_{SCC}/\bar{\gamma }$), PSC (${\bar{\gamma }}_{SC}/\bar{\gamma }$), SEC (${\bar{\gamma }}_{SEC}/\bar{\gamma }$), and SECps (${\bar{\gamma }}_{SECps}/\bar{\gamma }$) as functions of the multipath fading parameter (*m*) over Fisher–Snedecor F and Nakagami-*m* (*n* → ∞) fading channels, where $\bar{\gamma }$ = 5 dB and $\gamma_{\tau }$ = 5 dB.

In Figure 1, we show a comparison of the normalized average output SNR values of SCC (${\bar{\gamma }}_{SCC}/\bar{\gamma }$), PSC (${\bar{\gamma }}_{SC}/\bar{\gamma }$), SEC (${\bar{\gamma }}_{SEC}/\bar{\gamma }$), and SECps (${\bar{\gamma }}_{SECps}/\bar{\gamma }$) as functions of the multipath fading parameter (*m*) over Fisher–Snedecor F and Nakagami-*m* (*n* → ∞, Equations (18), (28), (82) and (84) in [53]) fading channels when $\bar{\gamma }$ = 5 dB and $\gamma_{\tau }$ = 5 dB. It can be seen from Figure 1 that the average SNR gain decreases gradually as *m* increases for various fading scenarios. Especially for the smaller values of *m*, this gain degrades quite rapidly. When one parameter varies and other parameters are fixed for comparison purposes, we find that: (*i)* the average SNR gain of the i.i.d. case ($\rho_{N}=0$ and/or $\;\rho_{G}=0$) is higher than that of the n.i.i.d. case ($\rho_{N}=0.5$ or $\rho_{G}=0.5$); (*ii)* the average SNR gain over Nakagami-m (*n* → ∞) fading channels is smaller than that over Fisher–Snedecor F (*n* = 5) fading channels; (*iii)* the average SNR gain of the PSC outperforms that of the SCC. Furthermore, it is clear that the average SNR gains of the SEC and the SECps (*L* = 3) become larger by comparing them with the dual-branch SSC, while the average SNR gain of the SECps outperforms that of the SEC. It is interesting to note that the average SNR gain of the SSC system in the n.i.i.d. case achieves the maximum value as $\gamma_{\tau }$ = 5 dB by comparing it with $\gamma_{\tau }$ = 8 dB (dot line) and $\gamma_{\tau }$ = 2 dB (dash line). This is because $\gamma_{\tau }$ = 5 dB approaches the optimum switching threshold $\gamma_{\tau }^{*}\approx$ 5.56 dB when $\bar{\gamma }$ = 5 dB, where $\gamma_{\tau }^{*}$ can be calculated with the help of the numerical analysis of (61).

**Figure 2:**
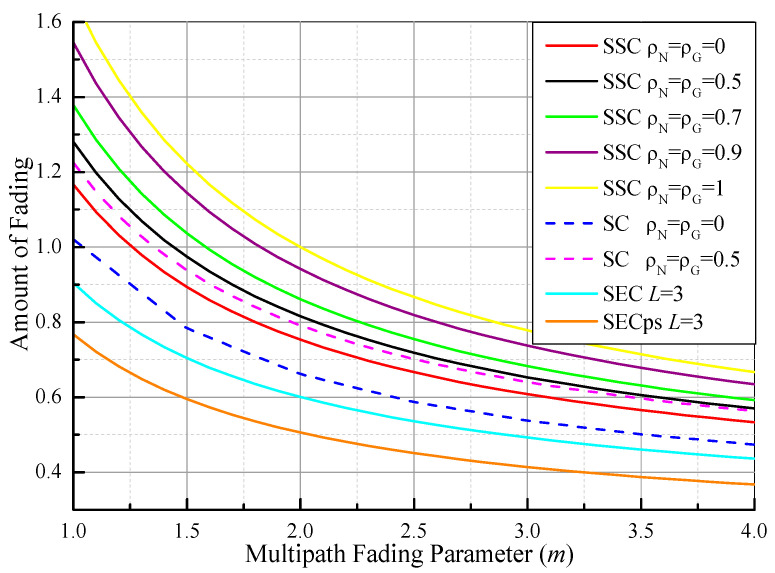
Comparison of the AoF values of different switched diversity systems as functions of the multipath fading parameter (*m*) over Fisher–Snedecor F fading channels, where *n* = 5, $\bar{\gamma }$ = 5 dB, and $\gamma_{\tau }$ = 5 dB.

Based on (67), Figure 2 illustrates the AoF values of switched diversity systems as functions of the multipath fading parameter (*m*) for various correlation conditions over Fisher–Snedecor F fading channels, where *n* = 5, $\bar{\gamma }$ = 5 dB, and $\gamma_{\tau }$ = 5 dB. As expected, it can be observed that the values of the AoF show similar behaviors as the average SNR gain values in Figure 1 as *m* increases. On the contrary, the AoF increases with the increase of the correlation coefficients ($\;\rho_{G}=\rho_{N}=0$, 0.5, 0.7, 0.9, 1). This is because the system performance degrades when the correlation coefficients become larger. For comparison purposes, the AoF values of the dual-branch PSC system, three-branch SEC, and SECps systems are also presented. It is evident that the AoF value of the PSC system has a lower value than that of SSC under the same correlation conditions, while the SECps system can provide better system performance than the SEC system.

**Figure 3:**
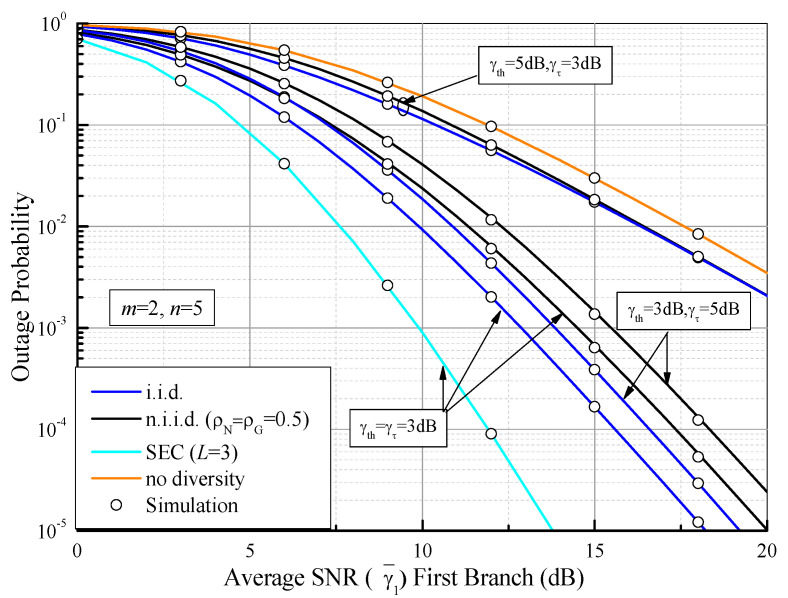
Outage probability values of dual-branch SSC and three-branch SEC systems as functions of the average SNR (${\bar{\gamma }}_{1}$) first branch over Fisher–Snedecor F fading channels, with different values of ${\bar{\gamma }}_{th}$ and $\gamma_{\tau }$ and with *m* = 2 and *n* = 5.

By using (26), (28) and (46), the OP values of SSC and SEC receivers are plotted in Figure 3 as functions of the average output SNR ($\bar{\gamma }$) first branch over F composite fading conditions. It can be seen that the OP achieves optimum performance as $\gamma_{th}$ = $\gamma_{\tau }$ and shows the worst performance as $\gamma_{th}$ > $\gamma_{\tau }$, regardless of the fading conditions. This is because the OP of the SSC receiver can be viewed as that of the PSC system as $\gamma_{th}$ = $\gamma_{\tau }$ in [53]. As expected, the OP of the SEC with *L* = 3 can be improved by comparing it with the dual-branch SSC system.

**Figure 4:**
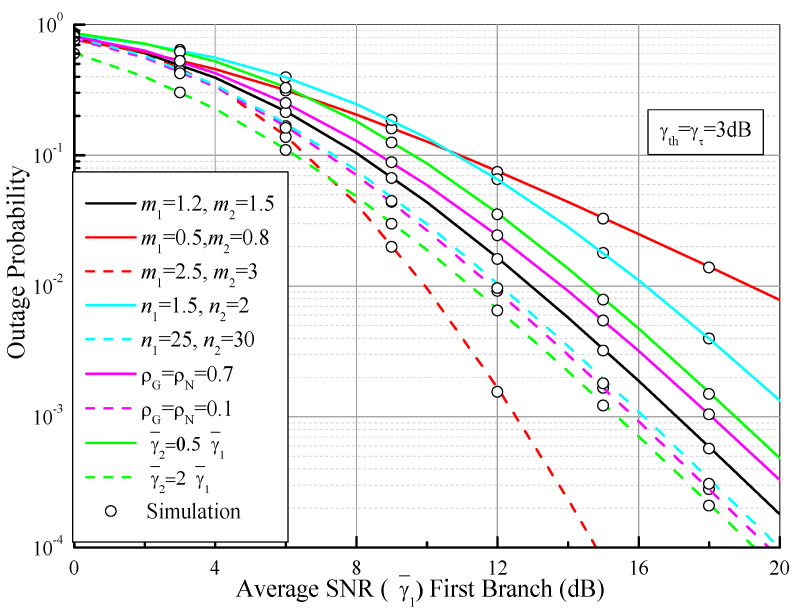
Outage probability values of dual-branch SSC systems as functions of the average SNR (${\bar{\gamma }}_{1}$) first branch over n.i.n.i.d. Fisher–Snedecor F fading channels, where ${\bar{\gamma }}_{th}$ = $\gamma_{\tau }$ = 3 dB.

Figure 4 depicts the OP of the dual-branch SSC system as a function of the average SNR (${\bar{\gamma }}_{1}$) of the first branch over n.i.n.i.d. Fisher–Snedecor F fading channels with ${\bar{\gamma }}_{th}$ = $\gamma_{\tau }$ = 3 dB. For comparison, a moderate fading case (*m*_1_ = 1.2, *m*_2_ = 1.5, *n*_1_ = 5, *n*_2_ = 6, $\rho_{G}$ = $\rho_{N}$ = 0.5, ${\bar{\gamma }}_{1}$ = ${\bar{\gamma }}_{2}$) is considered as a benchmark (black curve). As anticipated, it can be observed from Figure 4 that the OP is significantly improved when the multipath fading parameters (*m*) become larger and other parameters are fixed. On the other hand, when the correlation coefficients decrease, the shadowing parameters grow, or the average SNR of the second branch becomes larger than the one of the first branch, the OP is gradually raised. This is because the multipath fading parameters affect the curve slope of the OP, while the other parameters have an impact on the coding gain of the OP, where the coding gain is defined as the shifting degree of the OP curve to the left versus the SNR in a log–log scale.

**Figure 5:**
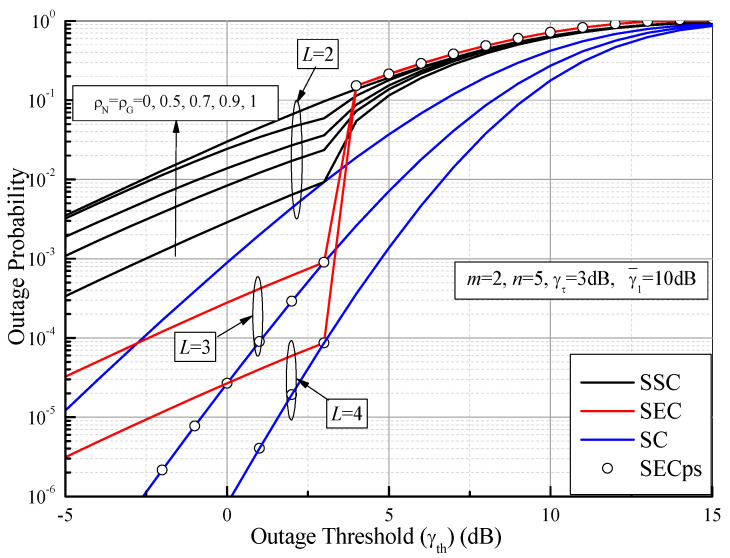
Outage probability values of different switched diversity systems as functions of the outage threshold ($\gamma_{th}$) over Fisher–Snedecor F fading channels with *m* = 2, *n* = 5, ${\bar{\gamma }}_{1}$ = 10 dB, and $\gamma_{\tau }$ = 3 dB.

Figure 5 plots the comparison of the OP values of different switched diversity systems as functions of the outage threshold ($\gamma_{th}$) with *m* = 2, *n* = 5, ${\bar{\gamma }}_{1}$ = 10 dB, and $\gamma_{\tau }$ = 3 dB. It is clear that the OP of the SSC greatly deteriorates with the increase of the correlation coefficients ($\rho_{G}=\rho_{N}=0$, 0.5, 0.7, 0.9, 1). These results demonstrate that the increase of the correlation coefficients leads to the worst received signals, while the OP of the SSC system tends toward that of a single branch (i.e., no diversity, $\rho_{G}$ = $\rho_{N}$ = 1). Importantly, as $\gamma_{th}\leq \gamma_{\tau }$, we can see from Figure 5 that the OP of the SEC significantly outperforms that of the SSC with the increase of the number of branches, while the OP of the SECps shows the same performance as that of the PSC; as $\gamma_{th}>\gamma_{\tau }$, the OP of all the switched diversity systems shows the worst value. This may be explained by the fact that the increase of the outage threshold value degrades the system performance and results in no diversity gain. Therefore, the switched diversity systems can provide the diversity gain only when $\gamma_{th}\leq \gamma_{\tau }$. These systems achieve the same performance with the PSC as $\gamma_{th}$ = $\gamma_{\tau }$.

**Figure 6:**
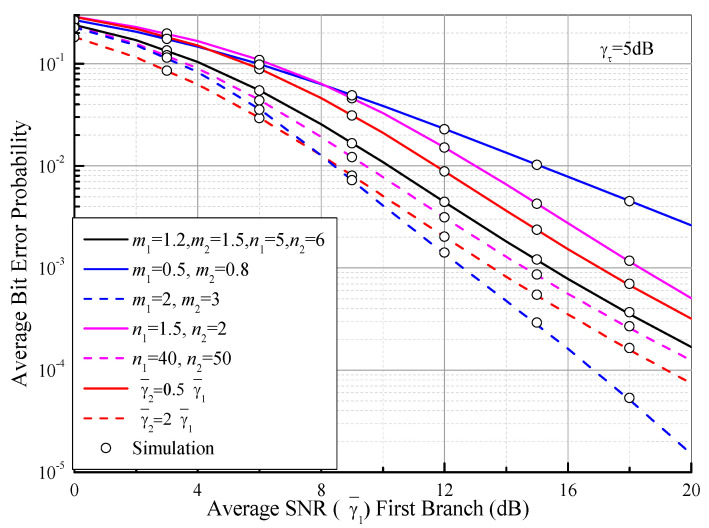
ABEP of DPSK for the dual-branch SSC as a function of the average SNR of the first branch (${\bar{\gamma }}_{1}$) over i.n.i.d. Fisher–Snedecor F fading channels with $\gamma_{\tau }$ = 5 dB.

Based on (35) and (68), Figure 6 plots the ABEP of DPSK for the dual-branch SSC receiver as a function of the average SNR of the first branch (${\bar{\gamma }}_{1}$) over i.n.i.d. Fisher–Snedecor F fading channels with $\gamma_{\tau }$ = 5 dB. For the sake of comparison, a moderate fading case (*m*_1_ = 1.2, *m*_2_ = 1.5, *n*_1_ = 5, *n*_2_ = 6, ${\bar{\gamma }}_{1}$ = ${\bar{\gamma }}_{2}$) is also assumed as a benchmark (black curve). As expected, it can be observed from Figure 6 that similar results are drawn as those in Figure 4.

**Figure 7:**
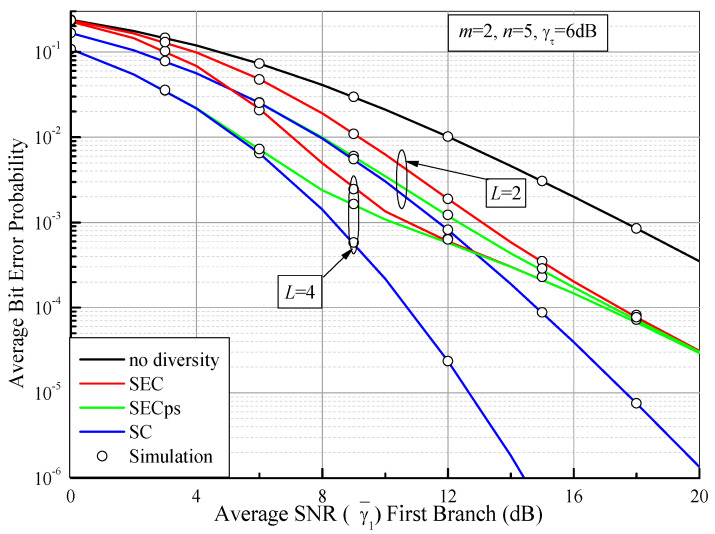
ABEP vaules of DPSK for the SEC and the SECps as a function of the average SNR of the first branch (${\bar{\gamma }}_{1}$) over i.i.d. Fisher–Snedecor F fading channels with *m* = 2, *n* = 5, and $\gamma_{\tau }$ = 6 dB.

By using (49), (58) and (68), Figure 7 depicts the comparison of ABEP values of DPSK for the SEC, the SECps, and the multibranch PSC as functions of the average SNR of the first branch (${\bar{\gamma }}_{1}$) over i.i.d. Fisher–Snedecor F fading channels with *m* = 2, *n* = 5, and $\gamma_{\tau }$ = 6 dB. We can see from Figure 7 that the ABEP of SEC with four branches outperforms that of the dual-branch SEC (which is equivalent to the dual-branch SSC [3]), while the ABEP of SECps is improved compared with the SEC in the low and middle SNR regions. This is because the SECps provides the same behaviors as the PSC in the low SNR region. However, the ABEP values of the SEC and the SECps gradually tend toward the same performance in the high SNR region. This can be explained by the fact that the switched diversity systems prefer to remain in one branch and no branch switching occurs when two or more branches have a higher SNR, which is adequate to provide the desired performance, than the switching threshold SNR.

**Figure 8:**
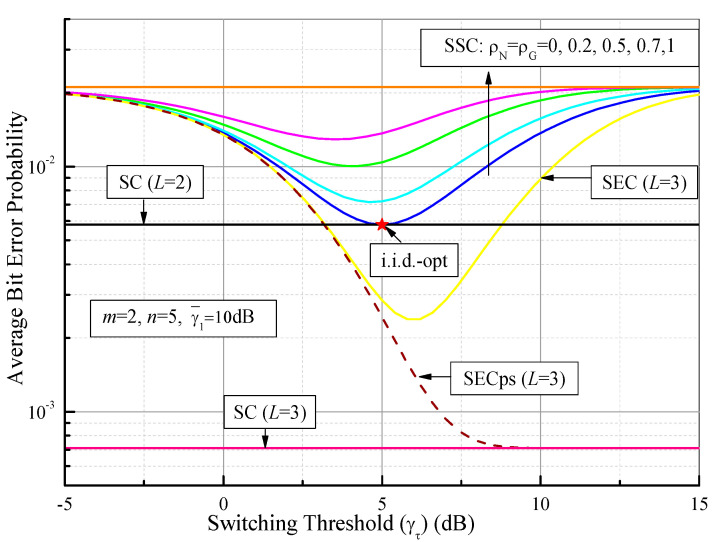
ABEP values of DPSK for the switched diversity systems as a function of the switching threshold ($\gamma_{\tau }$) over Fisher–Snedecor F fading channels with *m* = 2, *n* = 5, and ${\bar{\gamma }}_{1}$ = 10 dB.

**Figure 9:**
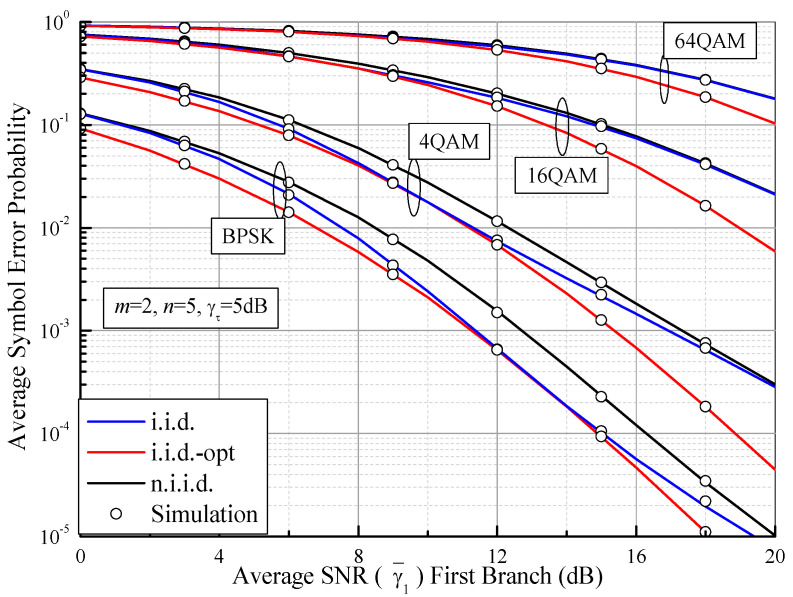
ASEP values of BPSK and MQAM for the SSC system as a function of the average SNR of the first branch (${\bar{\gamma }}_{1}$) over Fisher–Snedecor F fading channels with *m* = 2, *n* = 5, and $\gamma_{\tau }$ = 6 dB.

In Figure 8, we demonstrate the ABEP values of DPSK for SSC, SEC, and SECps as functions of the switching threshold ($\gamma_{\tau }$) over Fisher–Snedecor F fading channels, where *m* = 2, *n* = 5, and ${\bar{\gamma }}_{1}$ = 10 dB. It can be observed from Figure 8 that the ABEP values of the SSC and SEC are optimal when a certain optimal switching threshold is determined. As an example, the optimum value of the ABEP of DPSK for the SSC system in the i.i.d. case is Pe(*E*) = 0.005798, while the optimal switching threshold is $\gamma_{\tau }^{*}\approx$ 5 dB (i.i.d.-opt, red star). This optimal value is the same as the ABEP of PSC. Interestingly, the optimal ABEP of the SSC becomes larger and the optimal $\gamma_{\tau }^{*}$ becomes smaller when the correlation coefficient increases. Furthermore, when $\gamma_{\tau }$ becomes very small or is large enough that ${\bar{\gamma }}_{1}$ = 10 dB, the ABEP values of the SSC and the SEC gradually approach that of the no-diversity case ($\rho_{G}$ = $\rho_{N}$ = 1). These results show that all the branches are unavailable when $\gamma_{\tau }$ is higher, while all of the branches are acceptable when $\gamma_{\tau }$ becomes smaller, meaning the diversity system is likely to choose one branch and no longer provides diversity gain. The performance of the SECps gradually improves from SEC to PSC when $\gamma_{\tau }$ grows, and no optimal threshold exists. This is because no branch is acceptable and the SECps has to choose the branch with the largest SNR. In this case, although the error performance of the SECps is improved, the complexity of branch estimations increases. In addition, based on (74), (75) and (82), Figure 9 illustrates the ASEP values of BPSK and MQAM for dual-branch SSC as a function of the average SNR per symbol for i.i.d. (*m* = 2, *n* = 5, $\gamma_{\tau }$ = 5 dB) and n.i.i.d. ($\rho_{G}$ = $\rho_{N}$ = 0.5). At the same time, the optimum ASEP analysis (i.i.d.-opt) is also presented by using (76) and (83). It is clear that the ASEP performance of SSC improves as the average SNR grows and decreases with the increase of modulation order *M*.

**Figure 10:**
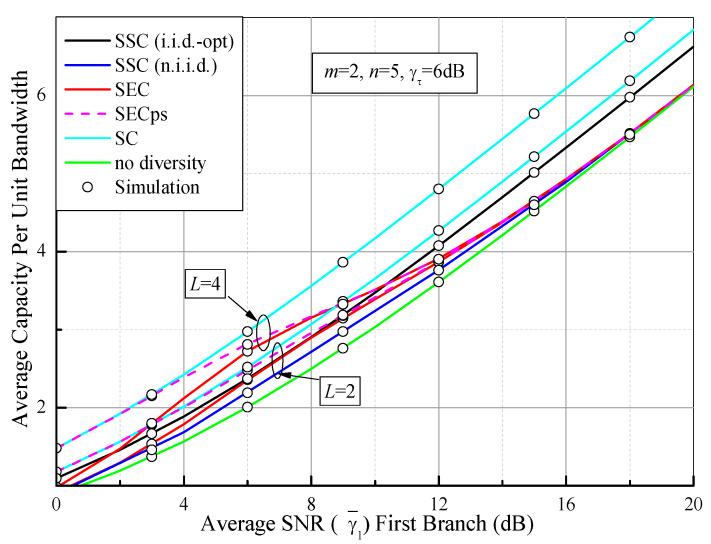
Comparison of the average capacity levels of different switched diversity systems as a function of the average SNR of the first branch (${\bar{\gamma }}_{1}$) over Fisher–Snedecor F fading channels with *m* = 2, *n* = 5, and $\gamma_{\tau }$ = 6 dB.

**Figure 11:**
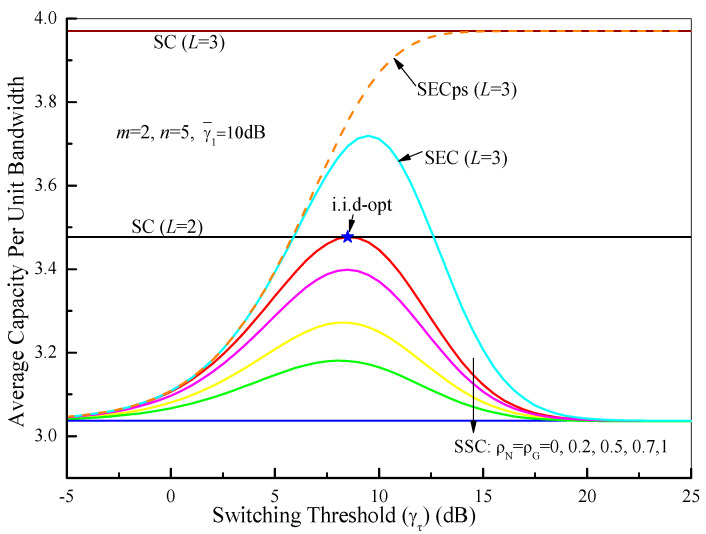
Comparison of the average capacity levels of different switched diversity systems as a function of the switching threshold ($\gamma_{\tau }$) over Fisher–Snedecor F fading channels with *m* = 2, *n* = 5, and ${\bar{\gamma }}_{1}$ = 10 dB.

Based on (90), (92), (93) and (94), the average capacity per unit bandwidth of the switched diversity systems as a function of the average SNR of the first branch (${\bar{\gamma }}_{1}$) over Fisher–Snedecor F fading channels with *m* = 2, *n* = 5, and $\gamma_{\tau }$ = 6 dB is depicted in Figure 10. As a comparison, the average capacities of the PSC (i.i.d.) and single-branch system (no diversity) are also shown. From Figure 10, it is evident that the average capacity for all of the cases improves with the increase of the average SNR. It is interesting that the average capacity of the dual-branch SSC receiver falls between the PSC and the single-branch system, while the SEC and the SECps show better performance only in the low and middle SNR regions and tend toward that of the single-branch system in the high SNR region. These results are similar to those shown in Figure 7. In Figure 11, we compare the average capacity per unit bandwidth of the switched diversity systems as a function of the switching threshold ($\gamma_{\tau }$), where *m* = 2, *n* = 5, and ${\bar{\gamma }}_{1}$ = 10 dB. It is observed that the optimum threshold for the maximum average capacity can be easily obtained by numerical analysis. These results in Figure 11 suggest the same conclusions as those in Figure 8. Therefore, it is necessary for the switched diversity systems to choose an appropriate switching threshold to balance the system performance and the branch estimations. Additionally, the switched diversity systems can provide the diversity gain in the low and middle SNR regions and have no distinct gain in the high SNR region compared with the single-branch system, which is helpful for enhancing the system reliability for wearable devices and IoT sensor nodes in low-power receiving scenarios.

## 7. Conclusions

In this paper, we presented a comprehensive performance analysis of SSC, SEC, and SECps schemes over Fisher–Snedecor F fading channels. We first studied the bivariate Fisher–Snedecor F distribution with arbitrary fading parameters, then the mathematical expressions of the statistical characteristics of the output SNR for the above schemes were deduced in various fading scenarios. In particular, certain novel analytical expressions of the statistical properties of the output SNR for the SECps scheme were obtained in terms of the multivariate Fox’s H-function. Thirdly, these performance metrics of interest, including the average SNR, AoF, OP, ABEP/ASEP, and average channel capacity, were investigated in detail for SSC, SEC, and SECps schemes under different fading conditions. Fourthly, the numerical and simulation results confirmed the validity of the theoretical expressions under various fading and shadowing scenarios. Finally, the obtained results suggested that the multipath parameter has a greater impact on the performance of SDC systems than the shadowing parameter, the correlation coefficient, or the average SNR. Moreover, the SDC systems can provide switched diversity gains only when the switching threshold is not too large or too small compared to the average SNR. These new results will be meaningful to enhance the system reliability in the design and deployment of future communication applications, including device-to-device, wearable communication, and Internet of Things. In future work, we will apply SDC techniques to design some wireless nodes for industrial Internet of Things applications, as two or more antennas can be installed on these nodes operating at 2.4 GHz or higher.

## Figures and Tables

**Table 1:** A list of SDC schemes used in some emerging wireless communications applications.

References	Research Topic	System Models	Fading Channels
[2]	Indoor off-body communication	SSC, SEC, and SECps	Nakagami-m
[18]	Outdoor wearable communication	SSC, SEC, and SECps	Nakagami-m
[19]	Ultra-ultraviolet communications	SSC	gamma–gamma
[20]	Multiuser ultraviolet communication	SSC	gamma–gamma
[21]	Low-power ultra-reliable low-latency downlink communications	PSC and SSC	Nakagami-m
[22]	Multichannel spectrum sensing	PSC and SSC	correlated Nakagami-m
[23]	Indoor millimeter wave communications	SSC and SEC	Rayleigh
[24]	Multiuser downlink wiretap networks	SECps	Nakagami-m
[25]	Mobile edge computing network	PSC and SSC	Nakagami-m
[26]	free space optical communications	distributed SSC	generalized Málaga and Gamma–Gamma
[27]	Multi-relay networks	SECps	shadowed Rician

**Table 2:** A list of acronyms used in this paper.

Acronym	Description	Acronym	Description
ABEP/ASEP	Average Bit/Symbol Error Probability	n.i.i.d.	Non-Independent and Identically Distributed
AoF	amount of fading	NCBFSK	non-coherent binary frequency shift keying
AWGN	additive white Gaussian noise	OP	outage probability
BDPSK	binary differential phase-shift keying	PDF	probability density function
CDF	cumulative distribution function	PSC	pure selection combining
i.i.d.	independent and identically distributed	SEC	switch-and-examine combining
i.n.i.d.	independent and non-identically distributed	SECps	SEC with post-examining selection
MGF	Moment-generating function	SDC	Switched diversity combining
MRC	maximum ratio combining	SNR	output signal-to-noise ratio
MQAM	M-ary quadrature amplitude modulation	SSC	switch-and-stay combining
n.i.n.i.d.	non-independent and non-identically distributed		

**Table 3:** Value of α under different numbers of branches of SEC when $\gamma_{\tau }^{*}=\alpha \bar{\gamma }$ over various fading conditions.

*L*	*α*		
	*m* = 2, *n* = 5	*m* = 2, *n* = 100	*m* = 2 (Nakagami-*m*) [50]
2	1	1	1
3	1.1874	1.1595	1.1582
4	1.3345	1.2790	1.2763
5	1.4571	1.3750	1.3710
6	1.5628	1.4554	1.4503
7	1.6564	1.5248	1.5185

## Data Availability

Not applicable.

## Appendix A

Based on (30), $MGF_{\gamma_{SSC}}^{\left(\gamma_{1}\right)}\left(s\right)$ can be written as:

(A1) $$MGF_{\gamma_{ssc}}^{\left(\gamma_{1}\right)}(s)=\underset{I_{A1}}{\underbrace{\int_{0}^{\infty }\exp(-s\gamma )g_{\gamma_{1}}(\gamma )d\gamma }}+\underset{I_{A2}}{\underbrace{\int_{\gamma_{\tau }}^{\infty }\exp(-s\gamma )f_{\gamma_{1}}(\gamma )d\gamma }}.$$

To solve the first integral *I_A_*_1_ in (A1), we insert (18a) into *I_A_*_1_ and use the identity in ([50], Equation (11)) to represent *exp*(−*x*) in terms of the Meijer G-function; with the aid of ([43], Equation (7.811.5)), *I_A_*_1_ can be derived as

(A2) $$\begin{array}{ll} I_{A1} & =\sum_{k=0}^{\infty }\sum_{l=0}^{\infty }\sum_{i=0}^{\infty }\sum_{j=0}^{\infty }\frac{\rho_{N}^{k+i}\rho_{G}^{l+j}\Theta \Gamma (\lambda_{1}){\left(m_{2}-m_{1}\right)}_{i}{\left(n_{2}-n_{1}\right)}_{j}\alpha_{1}^{m_{1}+k}\gamma_{\tau }^{m_{1}+k}}{k!l!i!j!\Gamma (m_{1})\Gamma (n_{1})\Gamma (m_{2}+k+i)\Gamma (n_{2}+l+j)(m_{1}+k)}\\ & \times G_{1,2}^{2,1}[s/\alpha_{2}|{}_{0,n_{2}+l+j}^{1-(m_{2}+k+i)}]{}_{2}F_{1}[\lambda_{1},m_{1}+k;1+m_{1}+k,-\alpha_{1}\gamma_{\tau }]. \end{array}$$

For the second integral *I_A_*_2_ in (A1), we use the primary definition of the Meijer G-function defined in ([43], Equation (9.301)) to find the closed-form solution. Firstly, substituting (1) into *I_A_*_2_, *I_A_*_2_ can be rewritten as:

(A3) $$\begin{array}{ll} I_{A2} & =\frac{\Lambda_{1}^{m_{1}}}{B(m_{1},n_{1})}\int_{0}^{\infty }\frac{\gamma^{m_{1}-1}\exp(-s\gamma )}{{\left(\Lambda_{1}\gamma +1\right)}^{m_{1}+n_{1}}}d\gamma -\frac{\Lambda_{1}^{m_{1}}}{B(m_{1},n_{1})}\int_{0}^{\gamma_{\tau }}\frac{\gamma^{m_{1}-1}\exp(-s\gamma )}{{\left(\Lambda_{1}\gamma +1\right)}^{m_{1}+n_{1}}}d\gamma \\ & =I_{A21}-I_{A22}. \end{array}$$

Similar to (3), *I*_A21_ can be obtained as:

(A4) $$I_{A21}=\frac{G_{1,2}^{2,1}[s/\Lambda_{1}|{}_{0,n_{1}}^{1-m_{1}}]}{\Gamma (m_{1})\Gamma (n_{1})}.$$

To find the closed-form expression of *I*_A22_, we represent (1 + *x*)*^a^* in terms of the Meijer G-function defined in ([51], Equation (10)), then apply the primary definition of the Meijer G-function, whereby *I*_A22_ can be rewritten as:

(A5) $$\begin{array}{ll} I_{A22} & =\frac{\Lambda_{1}^{m_{1}}}{\Gamma (m_{1})\Gamma (n_{1})}\int_{0}^{\gamma_{\tau }}\gamma^{m_{1}-1}G_{1,1}^{1,1}[\Lambda_{1}\gamma |{}_{0}^{1-m_{1}-n_{1}}]G_{0,1}^{1,0}[s\gamma |{}_{0}^{-}]d\gamma \\ & =\frac{\Lambda_{1}^{m_{1}}}{\Gamma (m_{1})\Gamma (n_{1})}{\left(\frac{1}{2\pi j}\right)}^{2}\int_{L_{1}}\int_{L_{2}}\left({ϒ}_{A}\right)\Gamma (t_{1})\Gamma (t_{2})\Gamma (m_{1}+n_{1}-t_{2}){\left(s\right)}^{-t_{1}}{\left(\Lambda_{1}\right)}^{-t_{2}}dt_{1}dt_{2}, \end{array}$$

where

(A6) $${ϒ}_{A}=\int_{0}^{\gamma_{\tau }}\gamma^{m_{1}-t_{1}-t_{2}-1}d\gamma =\frac{\gamma_{\tau }^{m_{1}-t_{1}-t_{2}}\Gamma (m_{1}-t_{1}-t_{2})}{\Gamma (m_{1}+1-t_{1}-t_{2})}.$$

By inserting (A6) into (A5), then employing the primary definition of the bivariate Meijer G-function again, the closed-form expression of *I_A_*_22_ can be obtained as:

(A7) $$I_{A22}=\frac{\Lambda_{1}^{m_{1}}\gamma_{\tau }^{m_{1}}}{\Gamma (m_{1})\Gamma (n_{1})}G_{1,1:0,1:1,1}^{0,1:1,0:1,1}[s\gamma_{\tau },\gamma_{\tau }\Lambda_{1}|{}_{-m_{1}}^{1-m_{1}}|{}_{0}^{-}|{}_{0}^{1-(m_{1}+n_{1})}].$$

Finally, substituting (A2), (A4) and (A7) into (A1), Equation (31a) can be obtained. Likewise, Equation (31b) can also be readily obtained.

## Appendix B

Let $x=sin^{2}\theta$ and dx=2sinθcosθdθ, then after some necessary changes of variables, Equation (73) can be written as:

(A8) $$P_{e}(E)=\frac{1}{2\pi }\int_{0}^{1}x^{-0.5}{\left(1-x\right)}^{-0.5}MGF_{\gamma_{SSC}}\left(g/x\right)dx.$$

By inserting (33) into (A8), Equation (A8) can be rewritten as:

(A9) $$\begin{array}{ll} P_{e}(E) & =\sum_{k=0}^{\infty }\sum_{l=0}^{\infty }\frac{\rho_{N}^{k}\rho_{G}^{l}\phi {\left(\gamma_{\tau }\eta_{2}\right)}^{m+k}{}_{2}F_{1}[\lambda ,m+k;1+m+k,-\eta_{2}\gamma_{\tau }]}{l!k!2\pi \Gamma (m)\Gamma (n)B(k+m,l+n)(m+k)}\Psi_{1}\\ & +\frac{\Psi_{2}}{2\pi \Gamma (m)\Gamma (n)}-\frac{\Lambda_{2}^{m}\gamma_{\tau }^{m}\Psi_{3}}{2\pi \Gamma (m\Gamma (n)}. \end{array}$$

where:

(A10a) $$\Psi_{1}=\int_{0}^{1}x^{-0.5}{\left(1-x\right)}^{-0.5}G_{1,2}^{2,1}[g/x\eta_{1}|{}_{0,n+l}^{1-(m+k)}]dx,$$

(A10b) $$\Psi_{2}=\int_{0}^{1}x^{-0.5}{\left(1-x\right)}^{-0.5}G_{1,2}^{2,1}[g/x\Lambda_{2}|{}_{0,n}^{1-m}]dx,$$

(A10c) $$\Psi_{3}=\int_{0}^{1}x^{-0.5}{\left(1-x\right)}^{-0.5}G_{1,1:0,1:1,1}^{0,1:1,0:1,1}[g\gamma_{\tau }/x,\gamma_{\tau }\Lambda_{2}|{}_{-m}^{1-m}|{}_{0}^{-}|{}_{0}^{1-(m+n)}]dx.$$

To obtain the closed-form expression of $\Psi_{1}$, similar to (A5), Equation (A10a) can be rewritten as:

(A11) $$\Psi_{1}=\frac{1}{2\pi j}\int_{L}\left(\Psi_{4}\right)\Gamma (t)\Gamma (n+l+t)\Gamma (m+k-t){\left(\frac{g}{\eta_{1}}\right)}^{-t}dt,$$

where $\Psi_{4}=\int_{0}^{1}x^{t-0.5}{\left(1-x\right)}^{-0.5}dx$. By using ([43], Equation (8.380.1)) and ([43], Equation (8.384.1)), $\Psi_{4}$ can be obtained as:

(A12) $$\Psi_{4}=B(t+0.5,0.5)=\frac{\Gamma (t+0.5)\Gamma (0.5)}{\Gamma (t+1)}.$$

By substituting (A12) into (A11) and employing the primary definition of the univariate Meijer G-function, the closed-form expression of $\Psi_{1}$ can be yielded as:

(A13) $$\Psi_{1}=\Gamma (0.5)G_{2,3}^{3,1}[g/\eta_{1}|{}_{0,n+l,0.5}^{1-(m+k),1}].$$

Likewise, the closed-form expression of $\Psi_{2}$ can be yielded as:

(A14) $$\Psi_{2}=\Gamma (0.5)G_{2,3}^{3,1}[g/\Lambda_{2}|{}_{0,n,0.5}^{1-m,1}].$$

Based on the definition of the bivariate Meijer G-function in ([45], Equation (13.1)), $\;\Psi_{3}$ can be expanded as:

(A15) $$\Psi_{3}={\left(\frac{1}{2\pi j}\right)}^{2}\int_{L_{1}}\int_{L_{2}}\left(\int_{0}^{1}x^{t_{1}-0.5}{\left(1-x\right)}^{-0.5}dx\right)\frac{\Gamma (m-t_{1}-t_{2})\Gamma (t_{1})\Gamma (t_{2})\Gamma (m+n-t_{2})}{\Gamma (1+m-t_{1}-t_{2})}{\left(g\gamma_{\tau }\right)}^{-t_{1}}{\left(\gamma_{\tau }\Lambda_{2}\right)}^{-t_{2}}dt_{1}dt_{2}.$$

By using the same method as (A13), the closed-form expression of $\Psi_{3}$ can be obtained as:

(A16) $$\Psi_{3}=\Gamma (0.5)G_{1,1:1,2:1,1}^{0,1:2,0:1,1}[g\gamma_{\tau },\gamma_{\tau }\Lambda_{2}|{}_{-m}^{1-m}|{}_{0,0.5}^{1}|{}_{0}^{1-(m+n)}].$$

Hence, by substituting (A13), (A14) and (A16) into (A9), the analytical expression in (74) can be obtained.

## Appendix C

Let $x=sin^{2}\theta$ and dx=2sinθcosθdθ, then after some necessary changes of variables, *I*_6_ can be written as:

(A17) $$\begin{array}{ll} I_{6} & =\sum_{k=0}^{\infty }\sum_{l=0}^{\infty }\frac{2c^{2}\rho_{N}^{k}\rho_{G}^{l}\phi {\left(\gamma_{\tau }\eta_{2}\right)}^{m+k}{}_{2}F_{1}[\lambda ,m+k;1+m+k,-\eta_{2}\gamma_{\tau }]}{l!k!\pi \Gamma (m)\Gamma (n)B(k+m,l+n)(m+k)}\Psi_{5}\\ & +\frac{2c^{2}}{\pi \Gamma (m)\Gamma (n)}\Psi_{6}-\frac{2c^{2}\Lambda_{2}^{m}\gamma_{\tau }^{m}}{\pi \Gamma (m)\Gamma (n)}\Psi_{7}, \end{array}$$

where:

(A18a) $$\Psi_{5}=\int_{0}^{0.5}x^{-0.5}{\left(1-x\right)}^{-0.5}G_{1,2}^{2,1}[g_{QAM}/x\eta_{1}|{}_{0,n+l}^{1-(m+k)}]dx,$$

(A18b) $$\Psi_{6}=\int_{0}^{0.5}x^{-0.5}{\left(1-x\right)}^{-0.5}G_{1,2}^{2,1}[g_{QAM}/x\Lambda_{2}|{}_{0,n}^{1-m}]dx,$$

(A18c) $$\Psi_{7}=\int_{0}^{0.5}x^{-0.5}{\left(1-x\right)}^{-0.5}G_{1,1:0,1:1,1}^{0,1:1,0:1,1}[g_{QAM}\gamma_{\tau }/x,\gamma_{\tau }\Lambda_{2}|{}_{-m}^{1-m}|{}_{0}^{-}|{}_{0}^{1-(m+n)}]dx.$$

Similar to (A11), $\Psi_{5}$ can be rewritten as:

(A19) $$\Psi_{5}=\frac{1}{2\pi j}\int_{L_{1}}\left(\Psi_{8}\right)\Gamma (t_{1})\Gamma (n+l+t_{1})\Gamma (m+k-t_{1}){\left(\frac{g_{psk}}{\eta_{1}}\right)}^{-t_{1}}dt_{1}.$$

where $\Psi_{8}=\int_{0}^{0.5}x^{t_{1}-0.5}{\left(1-x\right)}^{-0.5}dx$. By using ([43], Equation (8.391)) and ([54], Equation (06.19.26. 0010.01)), $\Psi_{8}$ can be expressed as:

(A20) $$\begin{array}{ll} \Psi_{8} & =\int_{0}^{0.5}x^{t_{1}-0.5}{\left(1-x\right)}^{-0.5}dx=B_{0.5}(t_{1}+0.5,0.5)\\ & =\frac{0.5^{t_{1}+0.5}}{\Gamma (0.5)}\left(\frac{1}{2\pi j}\right)\int_{L_{2}}\frac{\Gamma (0.5+t_{1}-t_{2})}{\Gamma (1.5+t_{1}-t_{2})}\Gamma (0.5-t_{2})\Gamma (t_{2}){\left(-0.5\right)}^{-t_{2}}dt_{2}. \end{array}$$

where $\;B_{x}\left(\cdot ,\cdot \right)$ is the incomplete beta function defined in ([43], Equation (8.391)). By inserting (A20) into (A19) and using the definition of the multivariable Fox’s H-function in terms of the multiple Mellin–Barnes-type contour integral defined in [44], after some mathematical manipulation, the closed-form expression of $\Psi_{5}$ in (A18a) can be obtained as:

(A21) $$\Psi_{5}=\frac{0.5^{0.5}}{\Gamma (0.5)}H_{1,1:1,2:1,1}^{0,1:2,1:1,1}[\frac{2g_{QAM}}{\eta_{1}},-0.5|{}_{\left(-0.5:-1,1\right)}^{\left(0.5:-1,1\right)}|{}_{\left(0:1),(n+l:1\right)}^{\left(1-(m+k):1\right)}|{}_{\left(0:1\right)}^{\left(0.5:1\right)}].$$

Similarly, the closed-form expressions of $\Psi_{6}$ and $\Psi_{7}$ can be respectively obtained as:

(A22) $$\Psi_{6}=\frac{0.5^{0.5}}{\Gamma (0.5)}H_{1,1:1,2:1,1}^{0,1:2,1:1,1}[\frac{2g_{QAM}}{\Lambda_{2}},-0.5|{}_{\left(-0.5:-1,1\right)}^{\left(0.5:-1,1\right)}|{}_{\left(0:1),(n:1\right)}^{\left(1-m:1\right)}|{}_{\left(0:1\right)}^{\left(0.5:1\right)}],$$

(A23) $$\Psi_{7}=\frac{0.5^{0.5}}{\Gamma (0.5)}H_{2,2:0,1:1,1:1,1}^{0,2:1,0:1,1:1,1}[2g_{QAM}\gamma_{\tau },\gamma_{\tau }\Lambda_{2},-0.5|{}_{\left(-0.5:-1,0,1),(-m:1,1,0\right)}^{\left(0.5:-1,0,1),(1-m:1,1,0\right)}|{}_{0}^{-}|{}_{0}^{1-(m+n)}|{}_{0}^{0.5}].$$

Finally, by substituting (A21), (A22) and (A23) into (A17), the analytical expression in (81) can be obtained.
